# Supplementary material for: ANKK1 Is a Wnt/PCP Scaffold Protein for Neural F-ACTIN Assembly
Source: Int J Mol Sci. 2024 Oct 4;25(19):10705. doi: 10.3390/ijms251910705 (PMC11477271; doi:10.3390/ijms251910705)
Supplement: Supplementary file 1 [file ijms-25-10705-s001.zip › ijms-3209969-supplementary.pdf]

International Journal of *Molecular Sciences*

MDPI

ANKK1 is a Wnt/PCP scaffold protein for neural f-actin assembly

Domínguez-Berzosa Laura, Cantarero Lara, Rodríguez-Sanz María, Tort Gemma, Garrido Elena, Troya-Balseca Johanna, Sáez María, Castro-Martínez Xóchitl, Fernandez-Lizarbe Sara, Urquizu Edurne, Calvo Enrique, López Juan Antonio, Palomo Tomás, Palau Francesc and Hoenicka Janet

Supplementary information: Materials and Methods, Figure S1 to S13, Table S1, S2, S3 and References

Materials and methods

1. Plasmids and Constructs

ANKK1: The GFP-tagged versions of the short ANKK1 polymorphic isoforms [ANKK1^{Thr239} (allele Thr239) and ANKK1^{Ala239} (allele Ala239)] were obtained by cloning the *ANKK1* cDNAs in the same reading frame as the pEGFP-N1 expression vector (Clontech, Mountain View, California, USA) as previously reported [1] The p.Ala239Thr polymorphic single nucleotide variations (SNV) is in linkage disequilibrium with the *Taq*IA. ANKK1^{Arg51} is a construct with a mutation in the kinase domain [1].

The V5-tagged full-length ANKK1 polymorphic isoforms constructs fl-ANKK1^{Thr239} (Haplotype 1 (H1) including Thr239 and A1), and fl-ANKK1^{Ala239} (Haplotype 2 (H2) including Ala239 and A2) were synthesized by Geneart AG (Regensburg, Germany). The mutant fl-ANKK1^{Arg51} was obtained by site-directed mutagenesis of fl-ANKK1 using the QuickChange XL Site-Directed Mutagenesis Kit (Stratagene, Santa Clara, California, USA) according to the manufacturer's protocol and verified by sequencing. The following primers were used for mutagenesis: 5′-GAGTACGCCATCAGGTGCGCCCCCTGC-3′ and 5′-GCAGGGGGCGCACCTGA

TGGCGTACTC-3′.

FARP1: The GFP-tagged version of FARP1 was a gift from Dr. Tomas Biederer (Tufts University, Boston, MA).

WGEF: The plasmid pRP[Exp]-CMV>EGFP(ns):hARHGEF19 (named pWGEF for simplicity) was purchased from Cyagen, California, USA and designed using the Vector Builder tool. This plasmid contains the GFP-tagged versions of the complete human cDNA of the *ARHGEF19* gene at the N-terminus.

MOCK: The plasmids used for non-overexpressing control cells were the pEGFP-N1 (Clontech, Mountain View, California, USA) and pcDNA3.1 (Invitrogen) vectors for GFP-tagged constructs (ANKK1^{Ala239}, ANKK1^{Thr239}, ANKK1^{Arg51} and FARP1) and V5-tagged constructs (fl-ANKK1^{Ala239} , fl-ANKK1^{Thr239,} and fl-ANKK1^{Arg51}) respectively.

Luciferase: Plasmids for luciferase reporter activity were the M50 Super 8x TOPFlash (with TCF/LEF binding motifs; a gift from Randall Moon, Addgene), 2xJun2TRE-TATA*TK*-LUC (with AP-1 binding motifs; a gift from Pierre Fafournoux), together with Renilla-Luc under the control of the HSV-thymidine kinase promoter as internal control (pRL-tk, Promega, Madison, WI, USA).

Knockdown: The *ANKK1* and *FARP1* shRNA constructs were obtained from Sigma-Aldrich (Saint Louis, Missouri, USA). The vectors targeted the following sequences: ANKK1-CCTGGGTATTGTGA TGGAGTT (Clone ID TRCN0000002302), FARP1-CTCATCTTACAGGCTCTATTT (Clone ID TRCN0000047245). The sequence for control shRNA was CAACAAGATGAAGAGCACCAA (SHC002).

The constructs are described in Table S1.

2. Human cell lines and generation of modified SH-SY5Y cell lines

We use human embryonic kidney HEK293T cells (ATCC Cat# CRL-3216, RRID:CVCL_0063) and neuroblastome SH-SY5Y cells (ATCC Cat# CRL-2266, RRID:CVCL_0019) with neuroblast-like morphology, positive for tyrosine hydroxylase (TH) and dopamine-β-hydroxylase, which can be differentiated to a mature neuron-like phenotype characterized by neuronal markers [2]. For transient transfections, HEK293T and SH-SY5Y cells were seeded at appropriate density in standard media and were transfected using Fugene HD (Promega, Madison, WI, USA) according to the manufacturer's instructions.

To generate stable overexpression (sc) cell lines, SH-SY5Y cells were transfected with pEGFP-N1, ANKK1^{Thr239}, pcDNA3.1, fl-ANKK1^{Thr239} or fl-ANKK1^{Arg51} plasmids using Fugene HD (Promega, Madison, WI, USA) and were cultured in standard media without selection antibiotic for two days. Then cells were selected by adding 500 μg/ml G418 (Sigma-Aldrich, Saint Louis, Missouri, USA) to the culture medium for three weeks. Positive transfected clones were

expanded and media with G418 was replaced every two days. Since the FARP1 plasmid does not contain an antibiotic resistance gene, the generation of FARP1 stable cell line SH-SY5Y was co-transfected with both FARP1 and pcDNA3.1 (which contains the neomycin-resistance gene). Cells were selected with G418 as described above and positive clones (GFP -expressing plus G418-resistant) were isolated and expanded. Positive clones were evaluated by western blot analysis.

For the generation of *ANKK1* and FARP1 knockdown stable SH-SY5Y cell lines (sh), three shRNA were tested for each gene (sequences are available on request). SH-SY5Y cells were transfected using Fugene HD (Promega, Madison, WI, USA) with shRNA vectors (clone ID and targeting sequences of the vectors that generated a successful knockdown at "plasmids" section). Two days after transfection selection medium containing 2 μg/ml puromycin (Sigma-Aldrich, Saint Louis, Missouri, USA) was added to generate a stable cell line. After three weeks of selection, clones were expanded and media containing puromycin was replaced every two days. Positive clones and knockdown efficiencies were evaluated by qRT-PCR.

The modified cell lines are described in Table S2.

3. Cell culture and SH-SY5Y lines differentiation

HEK293T cells were grown in DMEM (high glucose, Sigma-Aldrich, Saint Louis, Missouri, USA) supplemented with 10% (v/v) fetal bovine serum (FBS, Sigma-Aldrich), 2 mM L-Glutamine (Sigma-Aldrich) and 1% (v/v) Penicillin-Streptomycin (Sigma-Aldrich). SH-SY5Y cells were grown in DMEM/F12 (Sigma-Aldrich) supplemented with 10% (v/v) FBS (Sigma-Aldrich), 2 mM L-Glutamine (Sigma-Aldrich) and 1% (v/v) Penicillin-Streptomycin (Sigma-Aldrich).

Generated SH-SY5Y stable overexpressing cell lines, were grown in DMEM/F12 supplemented with 10% (v/v) FBS, 2 mM L-Glutamine, 1% (v/v) Penicillin-Streptomycin and 500 μg/ml G418. Generated SH-SY5Y knockdown ($_{sh}$) cell lines, were grown in DMEM/F12 supplemented with 10% (v/v) FBS, 2 mM L-Glutamine, 1% (v/v) Penicillin-Streptomycin and 2 μg/ml puromycin. All cells were maintained at 37ºC in a 5% CO_2 incubator.

For SH-SY5Y differentiation by starvation cells were washed three times with PBS and cultured in Dulbecco's Modified Eagles's Medium/Nutrient mixture F-12 (DMEM-F12) supplemented with 2 mm l-glutamine (Sigma-Aldrich), 100 mg/ml penicillin–streptomycin (Sigma-Aldrich) and serum-starved conditions (0% FBS) during 48 hours at 37◦C in a 5% CO2 incubator. For SH-SY5Y differentiation using retinoic acid (RA), cells were treated with RA 10 μM (Sigma-Aldrich) in DMEM/F12 with 1% FBS for 6 days, with every two days addition of fresh media with RA. For SH-SY5Y BDNF-differentiation, after 6 days of differentiation with RA, cells were subsequently treated with 50 ng/ml BDNF (PeproTech, Rocky Hill, NJ, USA) in neurobasal media supplemented with 1% B-27, 50 μg/ml gentamicin, 2 mM glutaMAX (Thermo Fisher Scientific, Waltham, MA, USA), 2 mM cAMP (Sigma-Aldrich) and 20Mm KCl.

4. Proteomic analysis

4.1. iTRAQ

Immunoprecipitated samples on beads were softly washed with NP-40 lysis buffer to minimize unspecific binding, and later, washed with Lysis Buffer without NP-40 for proteome analysis (Tris 50 mM pH 7.5, NaCl 150 mM, EDTA 5 mM, NaF 5 mM, Na3VO4 2 mM, DTT 1 mM, EDTA-free protease inhibitor mixture (Roche, Basilea, Switzerland)). After, beads were resuspended in Laemmli Buffer, heated at 95°C and ran on an SDS-PAGE gel (10% resolving gel and 4% stacking gel) at 20 mA/gel. The electrophoresis was stopped when the front dye has barely passed into the resolving gel, ensuring concentration of all proteins into a unique band. Protein staining was performed using colloidal Coomassie Brilliant Blue G-250 (Thermo Fisher Scientific, Waltham, MA, USA), and gel slices were subjected to in-gel-digestion by reduction with 40 mM DTT, followed by alkylation with 20 mM iodoacetamide and then digested overnight at 37ºC with modified pig trypsin (Promega, Madison, WI, USA) in 50 mM ammonium bicarbonate at a trypsin to protein ratio of 1:20. After desalting in C18 microcolumns, the resulting tryptic peptides were labeled with iTRAQ 4plex isobaric reagents with minor modifications [3]. Labeled peptides were loaded into the liquid chromatography/tandem mass spectrometry (LC-MS/MS) system. After, they were analyzed by using a C-18 reversed phase nano-column (75-mm inner diameter × 25 cm, 3-mm particle size, Acclaim PepMap 100 C18, Thermo-Fisher Scientific) in a continuous acetonitrile gradient consisting of 0–30% B in 145 min, 30–43% A in 5 minutes, and 43–90% B in 1 minute (A = 0.1% formic acid; B = 95% acetonitrile, 0.1% formic acid). A flow rate of 300 nl/min was used to elute peptides from the reverse phase nano-column to an emitter nanospray needle for real-time ionization and peptide fragmentation on an Orbitrap mass spectrometer (LTQ Orbitrap XL, Thermo Fisher Scientific). An enhanced resolution spectrum (resolution = 60,000) followed by the MS/MS spectra from the five most intense parent ions were analyzed during the chromatographic run (180

min). Dynamic exclusion was set at 40 seconds. For peptide identification, all spectra were analyzed with Proteome Discoverer (version 1.2, Thermo Fisher Scientific), using Sequest (Thermo Fisher Scientific; version 1.0.43.2).

For database searching, a human Uniprot database was interrogated selecting the following parameters: trypsin digestion with two maximum missed cleavage sites, precursor, and fragment mass tolerances of 20 ppm and 0.8 Da, respectively, carbamidomethyl cysteine, and iTRAQ modifications at N-terminal and Lys residues as fixed modifications, and methionine oxidation as a dynamic modification. For peptide and protein identification validation, results were loaded into Scaffold 3.0 software (Proteome Software Inc., Portland, OR), adding X!Tandem as an additional search engine. Peptide identifications were accepted if they could be established at greater than 95.0% probability as specified by the Peptide Prophet algorithm by Scaffold software. Quantitative results were manually checked in Proteome Discoverer for validating fragmentation spectra and sequence assignation.

4.2. Western blotting

Western blot has been performed following previous protocols. [4] Briefly, proteins were separated by 7,5-12,5% SDS-PAGE, transferred to nitrocellulose or PVDF membranes (GE Healthcare, Chicago, IL, USA), blocked (5% nonfat dry milk and 0.1% Tween 20 in Tris-buffered saline (TBS)) and incubated with primary antibodies overnight at 4ºC. The following antibodies were used: rabbit α-ANKK1 (1:500, orb2225758, Biorbyt, Cambridge, UK), rabbit α-StK2 (α-ANKK1, 1:1000 [5]), mouse α-FARP1 (1:500, H00010160-M01, Abnova, Taipei, TW), mouse α-GFP (1:200, sc-9996, Santa Cruz Biotechnology, Dallas, Texas, USA), mouse α-V5 (1:500, V8012, Sigma-Aldrich, Saint Louis, Missouri, USA), mouse α-RhoA (1:200, ARH05, Cytoskeleton, Denver, CO, USA), mouse α-Cdc42 (1:250, #ACD03-S, Cytoskeleton), rabbit α-RAC1 (1:500, ab97732, Abcam, Netherlands, Amsterdam), mouse α-RAC1 (1:500, #ARC03, Cytoskeleton), mouse α-alpha-Tubulin (1:2000, T6199, Sigma-Aldrich, Saint Louis, Missouri, USA), and mouse α-beta-ACTIN (1:8000, A5316, Sigma-Aldrich). All washing steps were performed with TBS-0.1% Tween 20. Primary antibodies were detected using goat α-mouse (1:10000), goat α-rabbit (1:10000), or goat α-rat (1:5000) antibodies coupled to HRP for 1 hour (Thermo Fisher Scientific). Proteins were processed for chemiluminescence with Amersham ECL Prime Western Blotting Detection Reagent (GE Healthcare, Chicago, IL, USA) and visualized by iBright™ CL1000 Imaging System (Thermo Fisher Scientific).

For RAC1 and RhoA western blot analysis, total protein levels were assessed using the Amersham QuickStain Protein Labeling kit (GE Healthcare, Chicago, IL, USA). Prior to blotting, samples were incubated 0.05% (v/v) with Cy™5 for 30 min at room temperature. Finally, the overall protein content was visualized with the Odyssey® CLx 9140 Imaging System (Li-Cor, Lincoln, NE,USA).

4.3. Immunoprecipitation

Cultured cells were homogenized in NP-40 lysis buffer [Tris 50 mM pH 7.5, NaCl 150 mM, 1% NP-40, EDTA 5 mM, NaF 5 mM, Na_3VO_4 2 mM, DTT 1 mM, EDTA-free protease inhibitor mixture (Roche, Basilea, Switzerland)] and precleared by centrifugation for 20 minutes at 13.000g. 1 mg of total lysate was incubated with the specific antibody (rabbit α-GFP, ab6556 (Abcam) ; rabbit α-V5, V8137 (Sigma-Aldrich); or rabbit α-FARP1, ab236948 (Abcam)) for 6 to 8 hours at 4ºC followed by incubation with Protein G Sepharose™ 4 Fast Flow (GE, Healthcare, Chicago, IL, USA) overnight at 4°C. Beads were softly washed with lysis buffer, resuspended in Laemmli Buffer, heated at 95°C and analyzed by SDS-Page and western blot.

5. Immunofluorescence

Cells were seeded on glass coverslips and fixed with PHEM buffer (Pipes 60 mM, Hepes 25 mM, EGTA 5 mM, MgCl 1 mM) supplemented with 0.25% glutaraldehyde, 4% paraformaldehyde and 4% sucrose. Next, cells were treated with a blocking solution (5% normal goat serum, 1% BSA (Sigma-Aldrich, Saint Louis, Missouri, USA)) in PBS and then incubated overnight with primary antibodies. ANKK1 was detected with rabbit polyclonal α-STK2 (1:200, generated by our group [5]). F-ACTIN was detected with the fluorescence conjugated Phalloidin-TRICT (P1951, Sigma-Aldrich). The following primary antibodies were used: mouse α-beta III tubulin (1:1000, Abcam), mouse α-FARP1 (1:200, H00010160-M01, Abnova, Taipei, TW), mouse α-GFP (1:200, sc-9996, SantaCruz), rabbit α-GFP (1:3000, ab6556, Abcam), rabbit α-V5 (1:1000, V8137, Sigma-Aldrich). Primary antibodies were detected using goat α-mouse or goat α-rabbit antibodies coupled to Alexa Fluor 488 or Alexa Flour 594 for 90 minutes (1:500, Thermo Fisher Scientific). All samples were mounted with DAPI Fluoromount-G (Thermo Fisher Scientific, Waltham, MA, USA) to visualize nuclei. Coverslips were then fixed on microscope slides.

6. Proximity ligation assay (PLA)

All antibodies used for this assay were before rigorously tested with immunofluorescence rabbit α-StK2 (1:200 [5]), goat α-ANKK1 (1:100, Santa Cruz, Dallas, Texas, USA), mouse α-FARP1 (1:50, Novus Biologicals, Littleton, CO, USA), rabbit α-FARP1 (1:200, Abcam), mouse α-RAC1 (1:200, Cytoskeleton), rabbit α-RAC1 (1:100, Abcam), mouse α-RhoA (1:200, Cytoskeleton), rabbit α-RhoA (1:200, Abcam), mouse α-Cdc42 (1:200, Cytoskeleton) and rabbit α-WGEF (1:100, Novus Biologycals)]. PLA was performed according to the manufacturer's protocol (Duolink In Situ Red Starter Kit Mouse/Rabbit, Sigma-Aldrich, Saint Louis, Missouri, USA). Briefly, cells were seeded on glass coverslips, fixed with PHEM buffer, and permeabilized with 100% methanol for 20 minutes at -20ºC. Subsequently, cells were blocked with a blocking buffer for 1 hour at 37ºC. The primary antibodies were applied to the cells in diluent antibody buffer overnight at 4ºC in a humidity chamber. On the following day, cells were washed four times with Washing Buffer A (5 minutes each) and incubated with the plus and minus probes diluted at 1:5 in the diluent antibody buffer for 1 hour at 37ºC. Subsequently, cells were washed three times (5 minutes each) with Washing Buffer A and incubated with the ligase solution (1:40) for 30 minutes at 37ºC. Next, cells were washed twice with Washing Buffer A (2 minutes each) and then incubated with the polymerase solution (1:80) for 100 minutes at 37ºC. Following the amplification, cells were washed three times (10 minutes each) with Washing Buffer B followed by 1 minute wash with 0.01 Washing Buffer B. Cells were then covered with a DAPI containing mounting medium, and coverslips were then fixed on microscope slides. For combined PLA and immunofluorescence staining, cells were washed with 0.01 Washing Buffer B and then washed three times with PBS (5 minutes each). Immunofluorescences were performed as described below.

7. Luciferase reporter assays

For dual luciferase assays, 9 x 10^5 SH-SY5Y or 5 x 10^5 HEK293T cells were plated in P35mm plates and transfected with the ANKK1 and FARP1 expression vectors together with the reporter plasmids ("plasmid" section). 48 hours after transfection, cells were lysed and luciferase activity was measured using the Dual-Glo Luciferase Assay System (Promega, Madison, WI, USA). For TCF/LEF activity, as an internal control, cells were treated with 100 ng/ml WNT3A (R&D Systems) for 16 hours and luciferase activity was measured.

8. RNA isolation and quantitative RT-PCR analysis

Total RNA was isolated with Trizol reagent (Thermo Fisher Scientific, Waltham, MA, USA) following the manufacturer's instructions and using the Direct-zol RNA miniprep kit (ZymoResearch, Irvine, CA). After evaluating RNA integrity and concentration with a NanoDrop spectrophotometer (Thermo Fisher Scientific, Waltham, MA, USA), cDNA was synthesized from 1 µg of RNA using Maxima™ First-strand cDNA synthesis kit for real-time quantitative RT-PCR (Thermo Fisher Scientific, Waltham, MA, USA). cDNA was precipitated with 0.1 volumes of 3 M Sodium acetate and 2.5-3 volumes of ice-cold 100% ethanol at -20ºC overnight. Pellet was centrifuged at 13,000 rpm 4ºC for 30 min and washed twice with 0.5 ml ice-cold 75% ethanol. Finally, cDNA was resuspended to a final concentration of 100 ng/µL. Real-Time qPCR (RT-qPCR) experiments were performed in triplicate in a 384 plate Standard Real-Time PCR System with PowerUp™ SYBR™ Green Master Mix (Thermo Fisher Scientific, Waltham, MA, USA). cDNA was amplified using ANKK1 primers (forward: 5′-CAGCACATCGTGTCTATCTAC-3′ and reverse: 5′-CAGGCCGAAGTCTG AAATTTTG-3′) and FARP1 primers (forward:5′-GTACTGAGATGCTCCA TGAA-3′ and reverse: 5′-TTGAGTCAGGACTTGAAGTC-3′), with efficiency being evaluated by serial cDNA dilutions. The endogenous control GAPDH was used as a normalizing factor with primers (forward: 5′-CGGAGTCAACGGATTTGGTC-3′ and reverse: 5′-AATCATATTGGAACATG TAAACCATGTAGT-3′). The analyses were performed with 4% of DMSO using 200 ng of cDNA per reaction for ANKK1 amplification and 20 ng for FARP1 and GAPDH amplification. With the Quant Studio™ 6 Flex Real-Time PCR System (Thermo Fisher Scientific) the plate was subjected at 50ºC for 2 minutes, 95ºC for 10 minutes; followed by 40 cycles at 95ºC for 15 seconds, Temperature melting (Tm) (58ºC for ANKK1 and 56ºC for FARP1) for 30 seconds and 72ºC for 45 seconds followed by a melt curve step at 95ºC for 15 seconds, 60ºC for 1 minute and 95ºC for 15 seconds. Results were obtained with Quant Studio Software v1.3 (Thermo Fisher Scientific, Waltham, MA, USA) and using a standard curve method. [6]

9. Neuritogenesis assay

2 x 10^5 stable overexpressing SH-SY5Y cell lines were seeded on glass coverslips in 6-well plates and cultured in standard growth media (0h time point) or in the absence of serum for 48 hours to induce a differentiated state (48h time point). Cells were fixed with PHEM buffer at respective time points. For ANKK1 and FARP1 co-overexpressing cells, ANKK1 or fl-ANKK1 plasmids were transiently transfected in the FARP1-stable expressing cell line (scFARP1) and cells

were grown, fixed and imaged at the time points described above. Neurite/Cell area ratio and soma area per nuclei quantifications were performed as described in the "Image acquisition and processing" section.

10. *In vitro* scratch wound healing assay

5 x 10^5 SH-SY5Y cells were plated in a 12-well plate one day before transfection and the next day were transfected with appropriate plasmids. When cells reached 95 ~100% of confluency in standard media with serum, the medium was replaced with fresh serum-free media to inhibit further proliferation. At least 24 hours after transfection, the cell monolayer was scraped in a straight line to create a "scratch" with a p200 pipet tip. The wounding area was measured at 0, 22 and 45 hours after scratching, using the method described in the "Image acquisition and processing" section.

10. Image acquisition and processing

10.1. Western blotting

Band intensity in western blotting analysis was measured through the total area of the peaks using the ImageJ 1.45v software from the National Institutes of Health.

10.2. Quantification of Proximity ligation assay (PLA)

Images were captured using a HC x PL APO 63x/x1-2 oil immersion objective. The confocal pinhole was set to 1.0 Airy units. The original data was stored as 8-bit greyscale images with a spatial resolution of 1024×1024 pixels. Pixel sizes ranged from 0.09x0.09 µm to 0.18x0.18 µm. Z-stacks were acquired in 0.8 µm z-increments along with the cell thickness. Maximum-intensity projections of image stacks of protein interaction (red channel – PLA signal) and nuclei (blue channel-DAPI) were calculated. Image preprocessing for PLA dot segmentation involved two steps. First, median filtering (2D, 3-by-3 neighborhood around the corresponding pixel in the input image) was applied to reduce unstructured background noise. Subsequently, image contrast was enhanced by linear gray-level transformation. Contrast limits for the input image were calculated by saturating the bottom 0.8 quantile of all pixel values in the image. A segmentation algorithm based on Otsu's method was selected for the autonomous delimitation of PLA dots. Image artifacts were removed by morphological opening if they had an area < 0.24-0.54 µm^2.

Nuclei were also automatically detected. Before the application of any binarization algorithm, the image was processed with a median filter (2D, 3-by-3 neighborhood around the corresponding pixel in the input image) to reduce noise while trying to preserve edges. A thresholding operation returned a first rough binarization, identifying the nuclei present in the image. Non-homogeneous DAPI staining due to low DNA density areas can lead to the appearance of artefactual nuclei holes. These were removed by morphology operations: first, the total whole segments were obtained by subtracting the original binary image from the hole-filled (filling) image. Subsequently, a hole-free image was obtained by adding those segments smaller than a specified size (opening, ~ 7.5 µm^2) to the original binary image. By an area-based constraint, particularly small artifacts (>0.3*median nuclei area) were rejected from further consideration.

The dot/nuclei area ratio (calculated as the total dot area/ total nuclei area), instead of dots per cell, was the eligible parameter to quantify the PLAs. Avoiding underestimation of the protein interaction in some images presenting huge quantities of overlapping dots, was the reasoning behind this choice.

10.3. Colocalization analysis

For the analysis of ANKK1-FARP1/F-ACTIN colocalization in the growth cone, neurites and dendritic filopodia of RA-differentiated SH-SY5Y cells, images were captured using an HC x PL APO 100x/3.5 oil immersion objective. The confocal pinhole was set to 499.96 mAiry units. The original data was stored as 8-bit greyscale images with a spatial resolution of 1256×1256 pixels. Pixel sizes were 0.025x0.025. Z-stacks were acquired in 0.012µm z-increments along with the structure thickness.

For the analysis of ANKK1-Phalloidin colocalization at subcellular cell migration structures of undifferentiated SH-SY5Y cells, images were captured using an HC x PL APO 100x/1.4 oil immersion objective with 0.45/0.55 zoom. The confocal pinhole was set to 499.96 mAiry units. The original data was stored as 16-bit greyscale images with a spatial resolution of 832×832 pixels. Pixel sizes ranged from 0.025x0.025 to 0.031x0.031. Z-stacks were acquired in 0.12 µm z-increments along with the structure thickness.

For the analysis of ANKK1-WGEF colocalization in undifferentiated and RA-differentiated SH-SY5Y cells, images were captured using an HC x PL APO 100x/1.4 oil immersion objective with 0.75 zoom. The confocal pinhole was set to 999.97 mAiry units. The original data was stored as 8-bit greyscale images with a spatial resolution of 1024×1024 pixels. Pixel sizes were 0.152x0.152. Z-stacks were acquired in 0.8 µm z-increments along with the thickness of the cell. For the

detailed analysis of ANKK1-WGEF colocalization in dendrites, images were acquired using an HC PL APO CS2 100×/1.40/0.70 Oil objective and Hybrid detectors (HyD). Emission depletion was accomplished with a 592 nm and 660 nm STED laser. Images were captured separately in sequential scans. A white laser at the desired wavelength for each sample provided excitation. 1600×1600 pixel images were acquired with a pixel size of 22 nm and the confocal pinhole was set to 1 AU. Image deconvolution was performed with Huygens Professional Software v17.10.0p8 (SVI, Leiden, The Netherlands).

For all the experiments above, maximum-intensity projections of image stacks were calculated. Intensity profiles were obtained by plotting the averaged fluorescence intensity of both channels along a straight line of a certain thickness crossing the Region of interest (ROI). The scatter plots were generated by plotting the two intensity values for each pixel against each other.

For ANKK1–WGEF colocalization, protein segmentation was an essential component for the subsequent colocalization analysis. Binarization (threshold luminance value of 0.05) applied to the median filter (2D, 3-by-3 neighborhood) of both channels rendered binary masks delimitating the protein regions. Image post-processing included the area opening for removing artifacts (<75 µm). The resulting masks were applied to both channels to exclude those pixels outside the ROIs. Colocalization was then quantified by Pearson's correlation coefficient (PCC) and Manders' overall overlap coefficient (MOC). The PCC is a well-established measure of linear correlation, defined such that:

$$\mathrm{Pcc} = \frac{\sum\left(Ch1_i - \overline{Ch1}\right) \times \sum\left(Ch2_i - \overline{Ch2}\right)}{\sqrt{\sum\left(Ch1_i - \overline{Ch1}\right)^2 \times \sum\left(Ch2_i - \overline{Ch2}\right)^2}} ,$$

The Manders' coefficients are well-established co-occurrence (overlap of two images while taking into account pixel intensity) measures, which simply calculate the percentage of total signal from one channel that overlaps with signal from the other. Furthermore, Manders proposed an overall overlap coefficient, such that:

$$\mathrm{MOC} = \frac{\sum_{i=1}^{n} Ch1_i \times Ch2_i}{\sqrt{\sum_{i=1}^{n} Ch1_i^2} \times \sqrt{\sum_{i=1}^{n} Ch2_i^2}}$$

Here, $Ch2_i$ and $Ch2_i$ stand for the intensities at pixel *i* in the green and red/purple channel, respectively, and the horizontal bar denotes the average value in the corresponding channel.

10.4. Neuritogenesis assay

Images were captured using a HC x PL APO 63x/1 oil immersion objective. The confocal pinhole was set to 1.0 Airy units. The original data was stored as 12-bit greyscale images with a spatial resolution of 1024×1024 pixels. Pixel sizes were 0.018x0.018 µm. Z-stacks were acquired in 1 µm z-increments along with the cell thickness. Maximum-intensity projections of image stacks of F-ACTIN (red channel - Phalloidin-TRICT) and nuclei (blue channel-DAPI) were calculated.

A custom 2-stage unsupervised segmentation method was implemented to discriminate between the cell body and neurites regions. The first stage involved cell segmentation. First, F-ACTIN was identified from the background by modeling the pixel intensity distribution as a univariate Gaussian mixture (2 components). The Gaussian component with the highest mean intensity was identified as the one corresponding to the F-ACTIN network. Artifacts (objects<40 µm^2) were then removed by a morphological area opening operation. Subsequent morphological closing with a structuring element (disk of radius 5) fills the mistakenly imputed holes within some cell regions. A final edge smoothing operation provides the definitive binary cell mask, used as the input for the second stage. The second stage aimed to distinguish between cell soma and neurites. For that purpose, a Euclidean distance-based method capable of identifying structure thickness was used. [7] The approach started by identifying edges and their contour normal directions by the application of a sobel operator (3x3). Next, a line was traced from each pixel following the estimated direction to find all the intersection points. Euclidean distances with the intersection points were then calculated, keeping only the distance to the nearest intersected pixel. Cell bodies were subsequently defined by thresholding of the distance map with a minimum thickness (Euclidean distance=50 pixels; 0.9 µm). The next steps involved structure dilation with a disk-shaped structuring element of radius 5, followed by an area filtering operation for removing small objects (3.5 µm). Inward interpolation is then used to fill the regions specified by the initial foreground mask. Image binarization (Otsu's method) finally segmented cell somas. Neurites were identified from the difference between the initial binary mask and

cell somas. The neurite/Cell area ratio was calculated as the percentage of neurites area (number of pixels in the neurites mask) divided by total cell area (number of pixels in neurites + somas masks). F-ACTIN intensity levels were calculated as the mean grey levels of the red channel masked with the binary image obtained in the first stage of the segmentation method (cell area).

10.5. Dendritic filopodia analysis

Images were captured using an HC x PL APO 100x/1.4 oil immersion objective. The confocal pinhole was set to 0.8Airy units. The original data was stored as 16-bit greyscale images with a spatial resolution of1496×1496 pixels. Pixel sizes ranged from 0.026x0.026 µm. Z-stacks were acquired in 0.3 µm z-increments along with the cell structure thickness. Maximum-intensity projections of 3D image stacks of Phalloidin (red channel – F-ACTIN) were calculated.

A custom skeleton-based semiautomatic algorithm was used to recognize dendritic branches. The model first isolates the whole dendritic filopodia. Background noise reduction by Gaussian filtering (σ=1) followed by image thresholding by Otsu's segmentation was implemented. Objects smaller than 5000 pixels (µm^2) were considered artifacts and removed from the binary image. To reduce contour imperfections, image smoothing by convolution kernel (window size=20) was applied.

Once the optimum binary image was reached, a skeletonization process was carried out. Skeletonization aims to reduce foreground regions in a binary image to a 1-pixel-wide representation (skeleton) that largely preserves the extent and connectivity of the original region. Spurious branches are often produced during the skeletonization process. The most common reason for this event is border noise, where every little protrusion can give rise to a skeletal branch. To avoid this undesired phenomenon a post-processing 'pruning' step was applied. Thus, spurious branches (< 20-pixel length) were permanently removed.

Three types of points can be identified in a skeleton: normal points (with two neighboring pixels), representing the skeleton chain links, branch points (with more than two neighboring pixels), where three or more skeleton branches meet and finally, end-points (with one neighboring pixel), indicating the end of a skeleton chain. At this stage, the algorithm prompts the researcher to re-labeling the initial and final pixels of the complete skeleton structure (from endpoints to normal points). This is a key step in the main axis recognition process, preventing these segments to be labeled as branches. A 4-step iterative process was then applied to differentiate between branches and the main axis: 1) By removing the branch points, a trimmed skeleton with many smaller segments was obtained. 2) Those containing an endpoint were labeled as branches (subtracting the endpoints causes a longitude difference in those segments including an endpoint, which are recognized as branches). 3) Subsequent removal of the segments labeled as branches from the original skeleton renders a less branched skeleton. 4) Branchpoints and endpoints were again recalculated. This loop was repeated until no more endpoints are found (only the main skeleton axis remains). Finally, branches were identified as the subtraction of the main axis from the original skeleton. The number of branches (per dendrite area) and the branch longitude (total branch longitude divided by total skeleton longitude, %) were extracted as biomarkers of dendrite branching to allow comparisons between cell lines. Both branch number and longitude were corrected to reduce potential bias related to differences in dendrite size.

10.6. Analysis of wound area reduction

In vivo images were acquired using 4x objective on a Leica DM IL LED Fluo (Leica Microsystems, Wetzlar, Germany). A custom image analysis method was applied to automatically identify the cell-free gap for wound reduction quantification. Stages were the following: (1) The ROI containing the wound was manually delimitated. (2) The true color RGB image was converted to a 16-bit grayscale image. (3) Contrast was enhanced by saturating the bottom 1% and the top 1% of all pixel values. (4) 2D Gaussian filtering (σ =2) was applied to reduce image noise. (5) Standard deviation (std) of the 9-by-9 neighborhood around each image pixel revealed different texture regions (low values in the gap and high values in the cell-covered region). (6) Gap area was identified inside the ROI as a region with a local std lower than a third of the mean local std. The percentage of wound reduction at the specified time points throughout the assay was calculated as:

$$\mathrm{wrp} = \frac{\mathrm{Warea}_{t=(22\ or\ 45h)} - \mathrm{Warea}_{t=0}}{\mathrm{Warea}_{t=0}} \times 100\,,$$

where Warea represents the number of pixels in the identified gap region at the different time points (t=22 or 45h).

10.7. Fluorescence intensity analysis

Images were captured using an HC x PL APO 100x/1.4 oil immersion objective. The confocal pinhole was set to 0.9 Airy units. The original data was stored as 8-bit greyscale images with a spatial resolution of 1024×1024 pixels. Pixel sizes ranged from 0.152x0.152 µm. Z-stacks were acquired in 0.8 µm z-increments until the entire cell thickness was captured. For ANKK1 and FARP1 fluorescence intensity, maximum-intensity projections of FARP1 (red channel – Alexa fluor 594), ANNK1 (green channel – Alexa fluor 488), and DAPI (blue channel – nuclei) were calculated. For RAC1 and RhoA fluorescence intensity, maximum-intensity projections of RAC1 (red channel – Alexa fluor 594), RhoA (green channel – Alexa fluor 488), and DAPI (blue channel – nuclei) were calculated.

A custom automatic algorithm was designed for the automatic quantification of mean protein fluorescence quantification. The accurate measurement of mean fluorescence intensity in the ROIs required the previous segmentation of cell and nuclei contours. First, image contrast was enhanced by linear gray-level transformation of the sum of red and green channels (fluorescence intensity of both proteins was considered for cell body delimitation). Contrast limits for the input image were calculated by saturating the bottom 0.98 quantile of all pixel values in the image. After noise removal by median filtering (2D, 3-by-3 neighborhood around the corresponding pixel in the input image), the autonomous delimitation of cell contour was obtained by binarization with a threshold luminance value of 0.1. The morphological opening was the eligible technique for removing unwanted small artifacts (<75 µm) arising from the binarization process. Nuclei were then segmented as described in the previous section. The whole cell mask was taken into account for the measurement of ANKK1/FARP1 and RAC1 mean intensity. However, nuclei were previously subtracted for RhoA intensity quantification.

All the analyses were implemented and performed using the MATLAB R_2019-2022a software (The MathWorks Inc., Natick, MA, USA).

11. Statistical analysis

The analysis of the experimental results was performed blindly by an independent investigator. Sample sizes were estimated based on our previous experience with each technique. Outliers were excluded from the analysis. The experiments were performed at least three times. All experiments with biological replicates are indicated in the figure legends.

Statistical analyses were performed using R (3.5.1 version). Data are expressed as mean ± standard deviation (SD) and individual values are displayed as dots. The normality of data was assessed by the Kolmogorov-Smirnov test. In case of normal distribution, One-sample *t*-test, *t*-Student test, or ANOVA were applied. Following significant results, multiple comparisons were conducted with the Bonferroni test. The number of biological replicates, or number of cells counted as well as the specific test applied in each experiment is indicated in the corresponding figure legend. p values less than 0.05 were considered significant.

Supplementary Figures

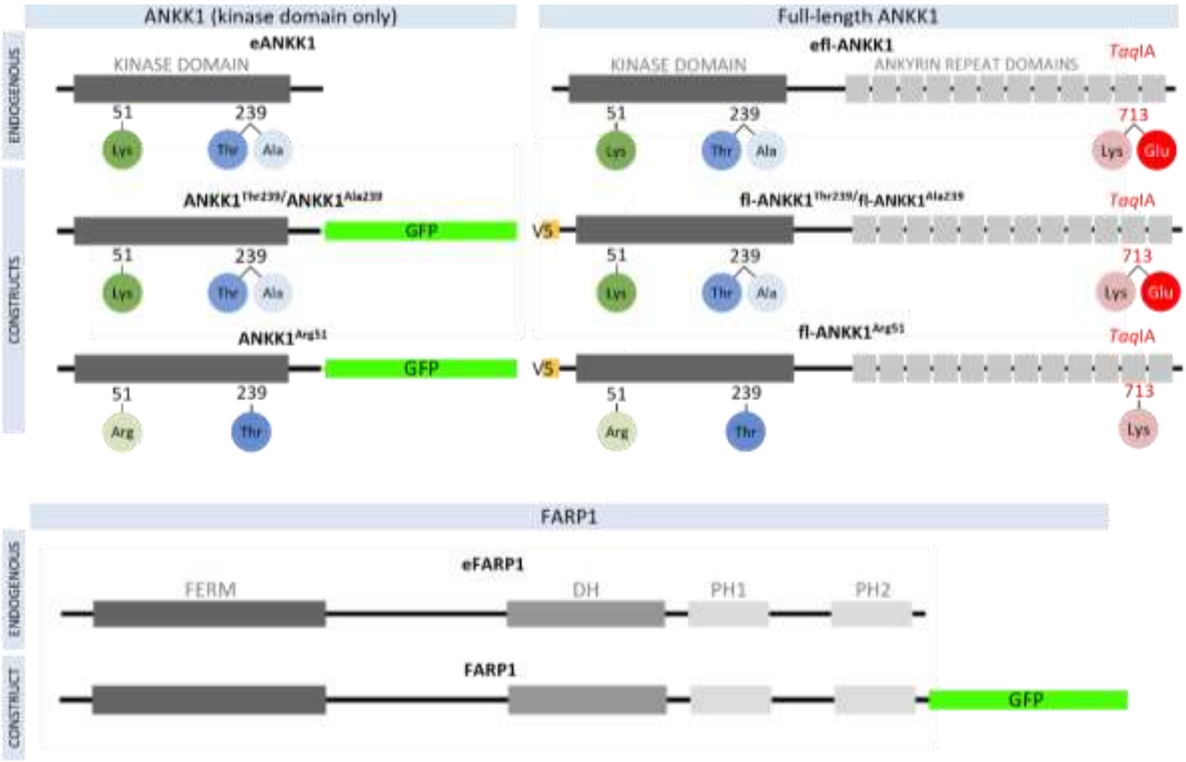

Figure S1. ANKK1 and FARP1 constructs used in this work. Upper panel: Functional domains of human endogenous ANKK1 isoforms [ANKK1-kinase (left) and full-length ANKK1 (right)] and recombinant proteins used in this study. The *Taq*IA *ANKK1* single nucleotide variation (SNV) (red bubbles) is in exon 8 and causes the substitution of Glutamic 713 by Lysine (p.Glu713Lys, red bubbles) in the eleventh ankyrin domain. *Taq*IA is in linkage disequilibrium with rs7118900, which changes Alanine 239 to Threonine (p.Ala239Thr, blue bubbles). ANKK1 polymorphic alleles were cloned in fusion with green fluorescence protein (GFP, short isoforms) or V5 for full-length isoforms. Other ANKK1 constructs include the change p.Lys51Arg (green bubbles). Lower panel shows the functional domains of endogenous human FARP1 and recombinant FARP1 protein in fusion with GFP used in this study. Abbreviations: DH: DBL-homology; FERM: 4.2, exrin, radixin, moesin; PH: pleckstrin homology.

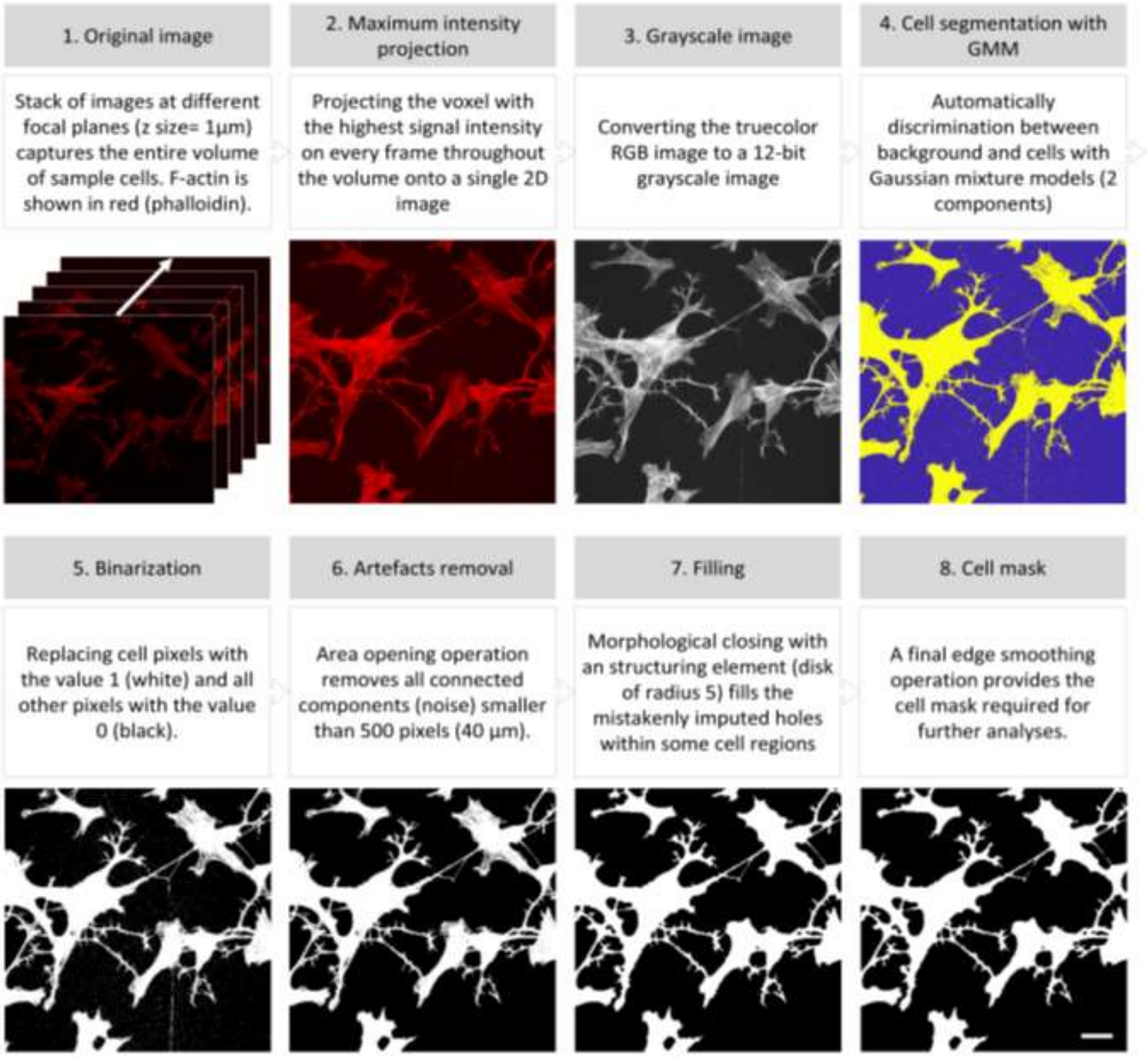

Figure S2. Overview of the automated cell segmentation workflow. Image segmentation creates a pixel-wise mask delimitating cell regions. The discrimination between cells and background is a key step forward in the quantification of both the neurites/somas areas and the fluorescence intensity. Scale bar: 25 µm.

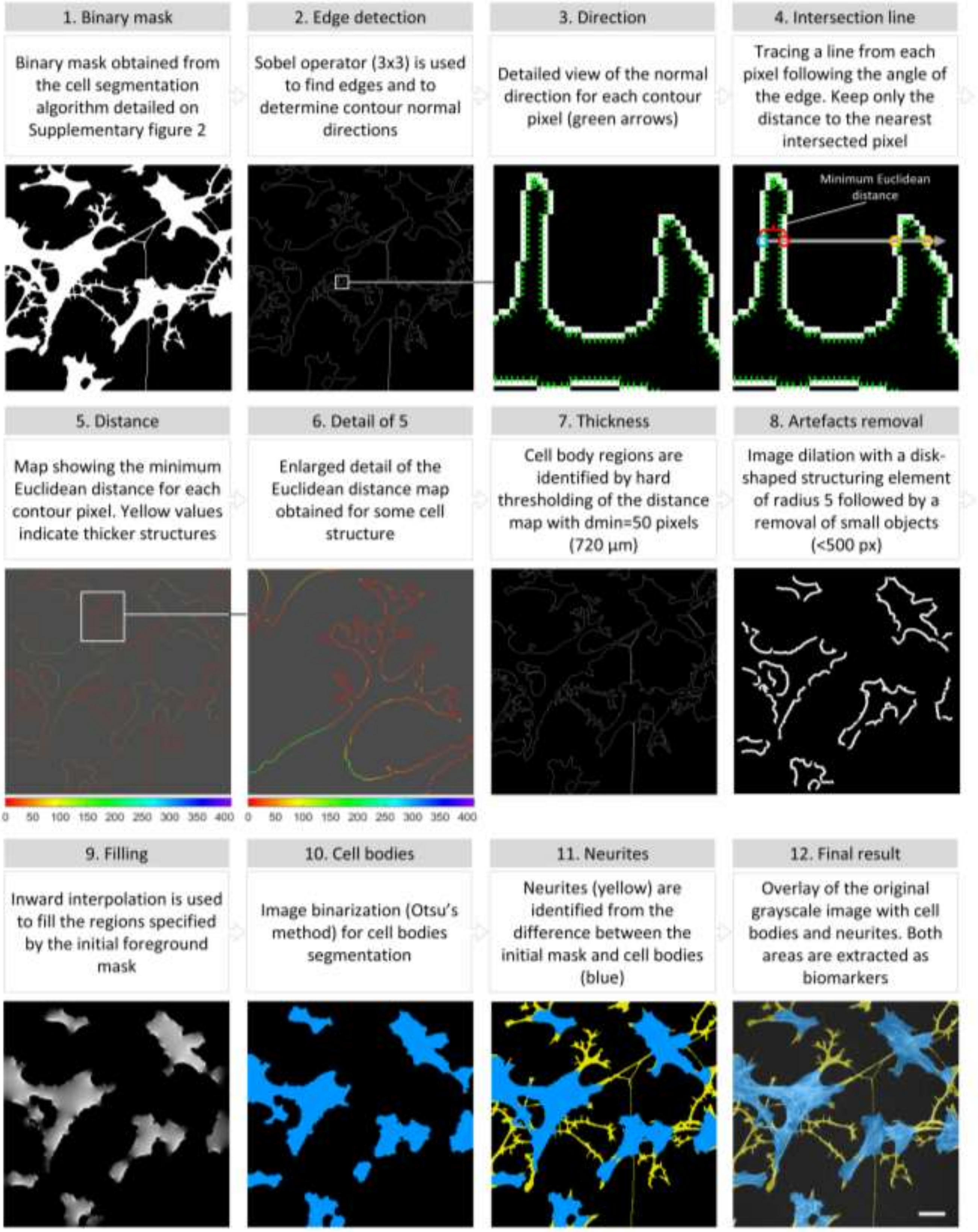

Figure S3. Workflow for the discrimination between neurites and soma regions. Starting with a previously generated cell mask, the algorithm automatically identifies soma and neurites regions on a cell, based on the structure thickness. The ratio neurites (yellow)/somas (blue) area is then computed and used as a marker of cell differentiation for group comparisons. Scale bar: 25 µm.

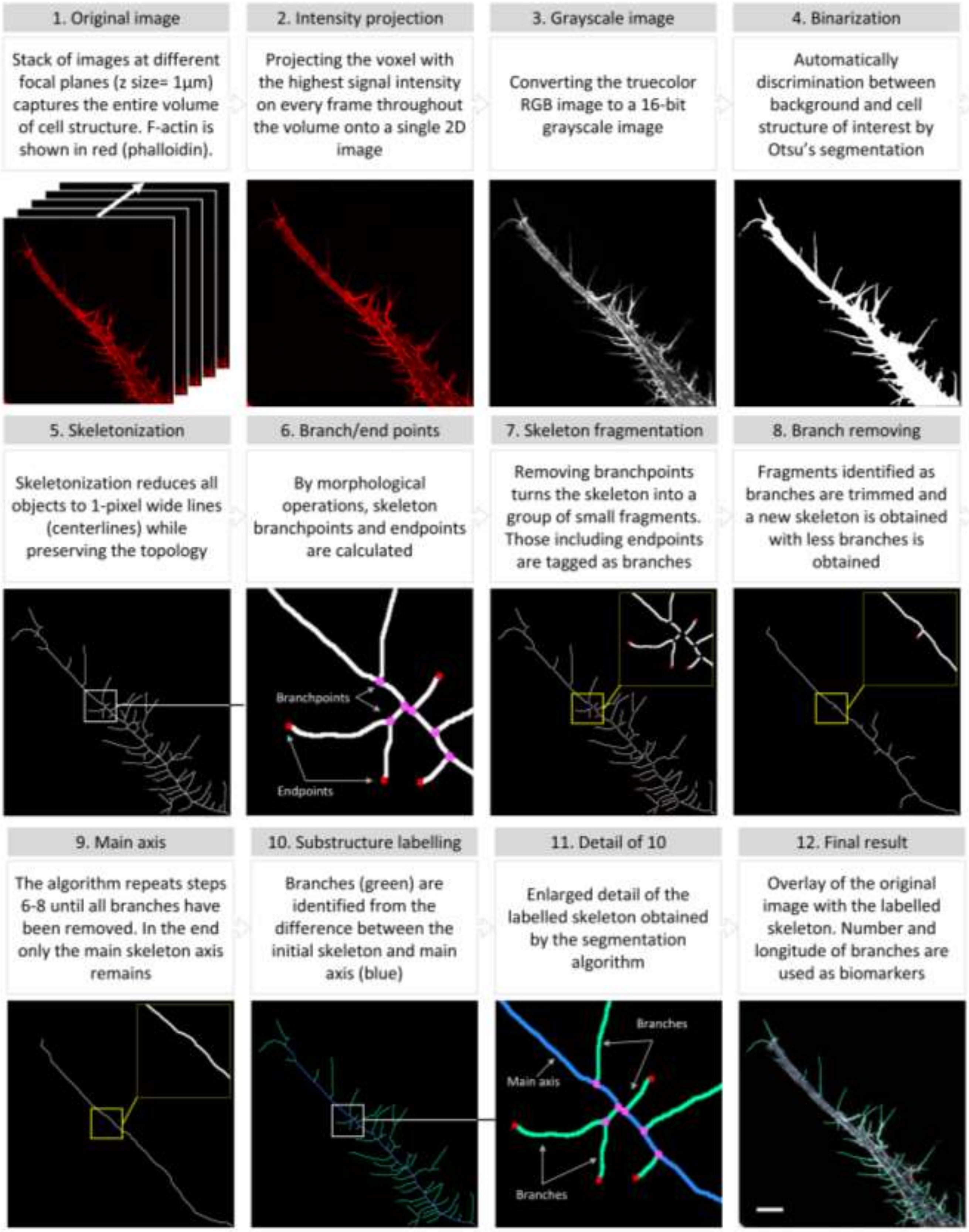

Figure S4. Detailed steps for the dendritic filopodia branching assessment. Our skeleton-based semi-automatic algorithm can recognize and label dendritic filopodia branches. Useful biomarkers such as branch number and longitude can be thus extracted, allowing comparisons to be made between the analyzed cell lines. Scale bar: 5 µm.

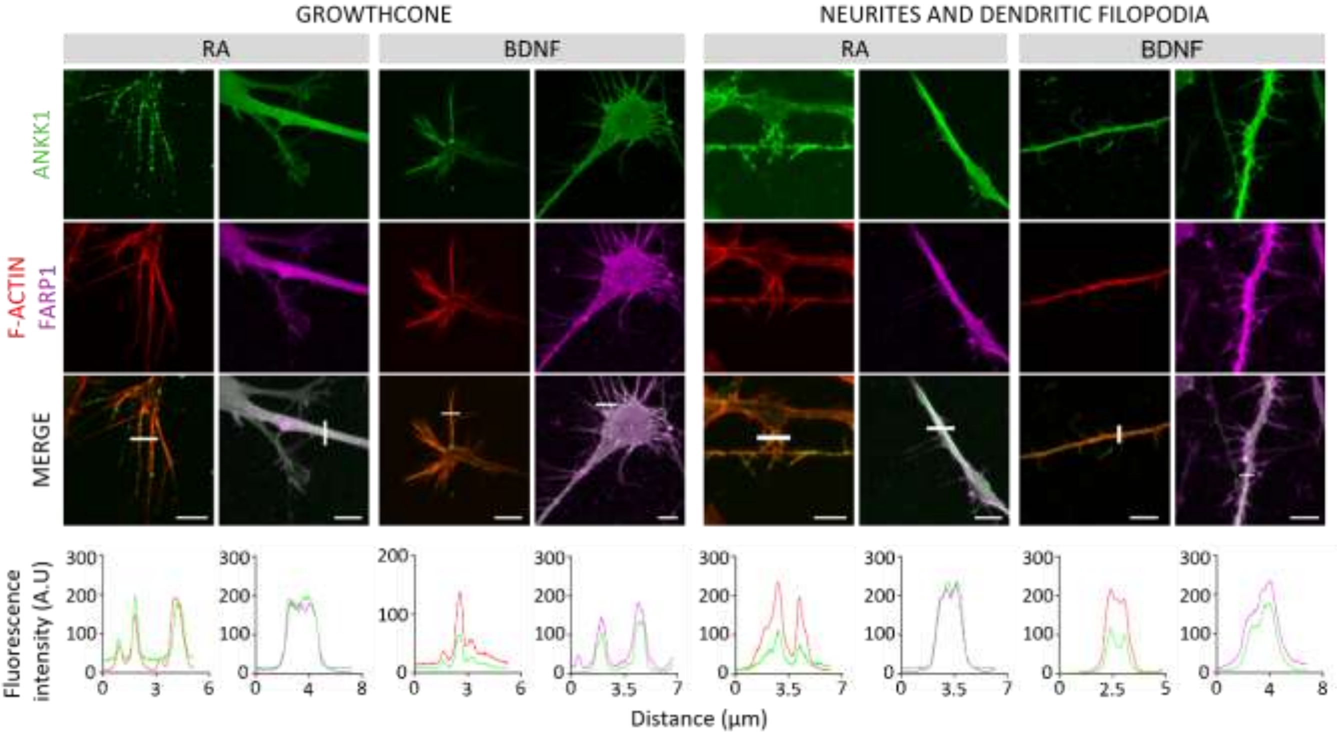

Figure S5. ANKK1 and FARP1 colocalize in neuritic structures rich in F-ACTIN. Representative confocal images of ANKK1 and F-ACTIN or FARP1 colocalization, in the growth cone (left) or neurites and dendritic filopodia (right) in RA or BDNF treated SH-SY5Y cells. Samples were stained with α-ANKK1 (StK2), α-FARP1 or Phalloidin. Maximum intensity projections are shown. Scale bar: 6 µm. The line profile plots indicate the intensity distribution of the green and red/purple channels along the white straight line crossing the ROI. Abbreviations: A.U: Arbitrary units; BDNF: Brain-derived neurotrophic factor; RA: Retinoic acid; ROI: Region of interest; U: Undifferentiated,.

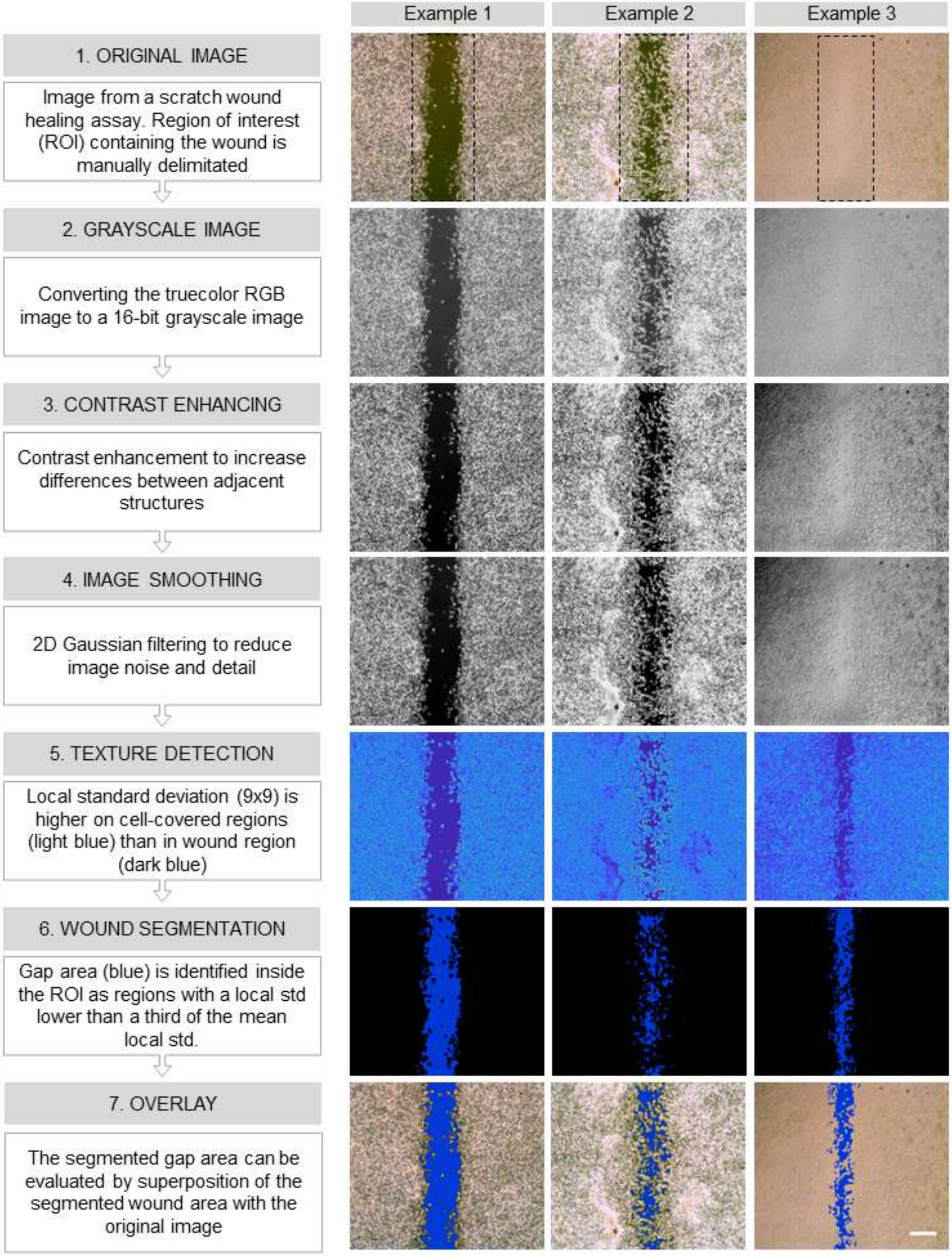

Figure S6. Schematic workflow and examples of intermediary steps for the automatic quantification of scratch wound healing assay. The flow diagram aims to automatically discriminate the cell-free gap from the background containing cells to evaluate the wound area. The examples provided illustrate how this process successfully delimitates the gap area both in images with continuous (Example 1) and patchy wound regions (Example 2) and even in those images with indistinguishable intensities between the gap and area containing cells (Example 3).

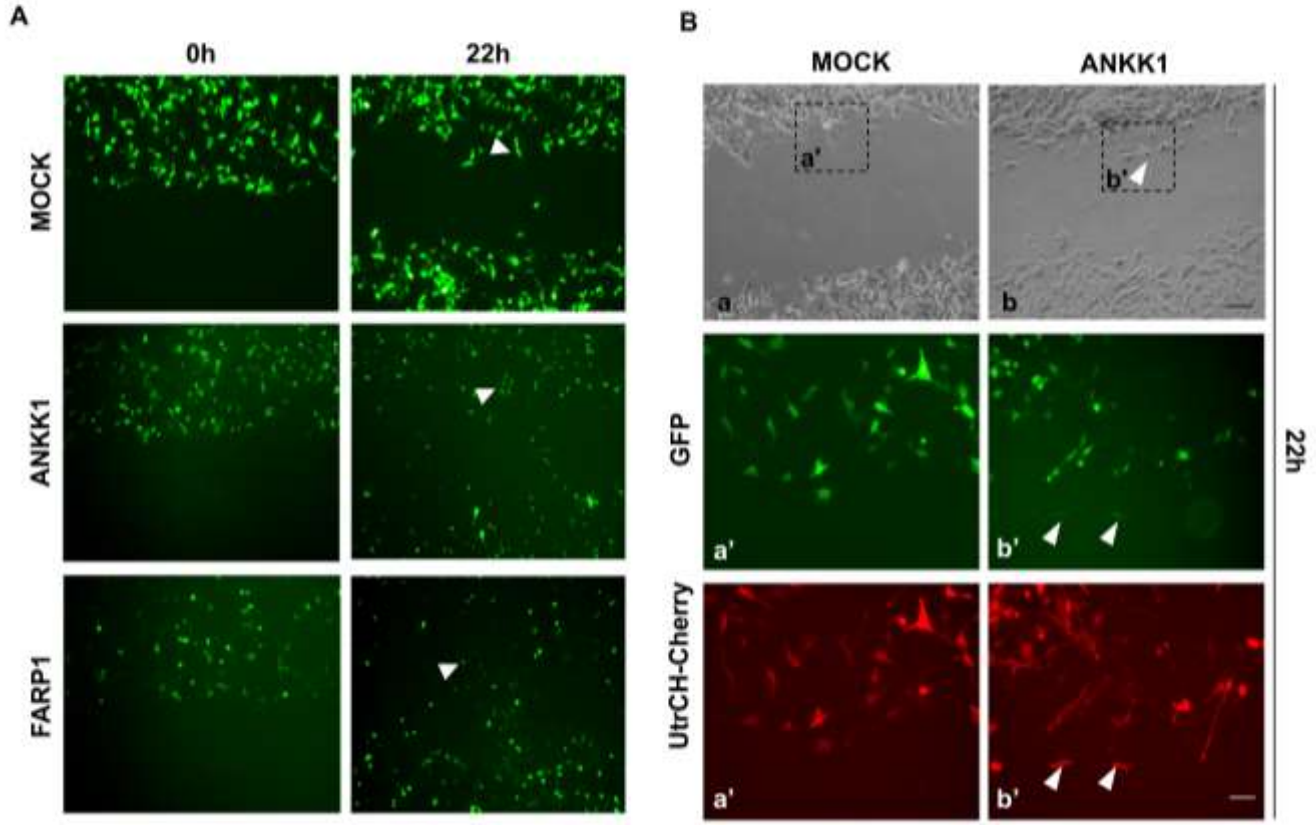

Figure S7. *In vivo* visualization of migrating cells (arrows) overexpressing either ANKK1 or FARP1 in scratch wound healing assays. **A**. Representative images of *in vivo* monitoring GFP signal in SH-SY5Y cells transfected with ANKK1 or FARP1 at 0 hours and 22 hours after scratching. Scale bar: 100μm. **B**. Representative images of phase contrast (a, b) and GFP/UtrCH-Cherry co-expression (a′, b′) showing a migrating leading front in SH-SY5Y cells transfected with ANKK1. The white arrows highlight the migrating cells closing the gap. Scale bar: (a, b) 100μm; (a′, b′) 40 μm.

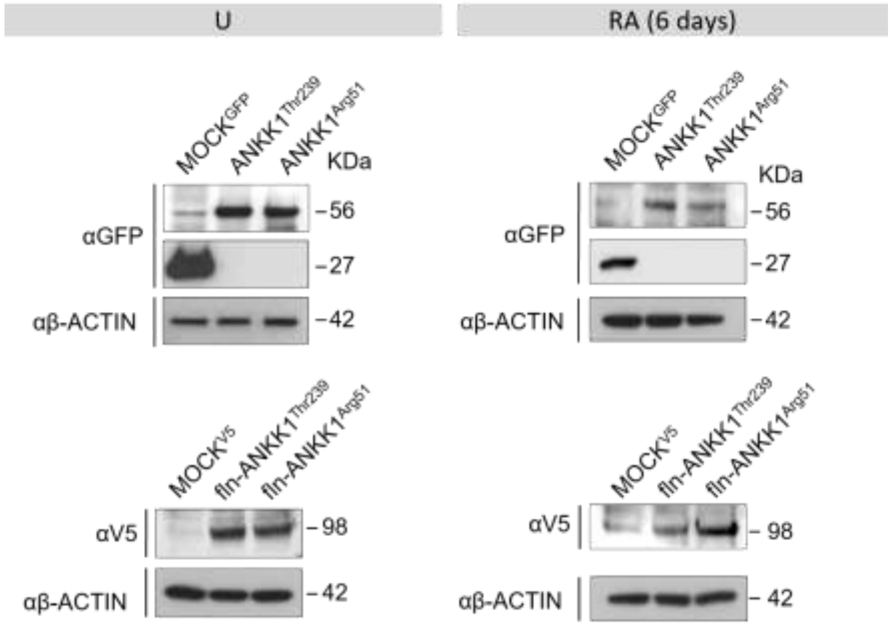

Figure S8. Transfection controls of the Wnt/PCP study by Western blot. Western blot analysis to monitor the transfected constructs in undifferentiated (U) and RA-differentiated (RA) SH-SY5Y cells. ANKK1 and ANKK1^{Arg51} constructs were detected with α-GFP; fl-ANKK1 and fl-ANKK1^{Arg51}. Constructs were detected with α-V5 (n=2). Abbreviations: RA: Retinoic acid; U: Undifferentiated.

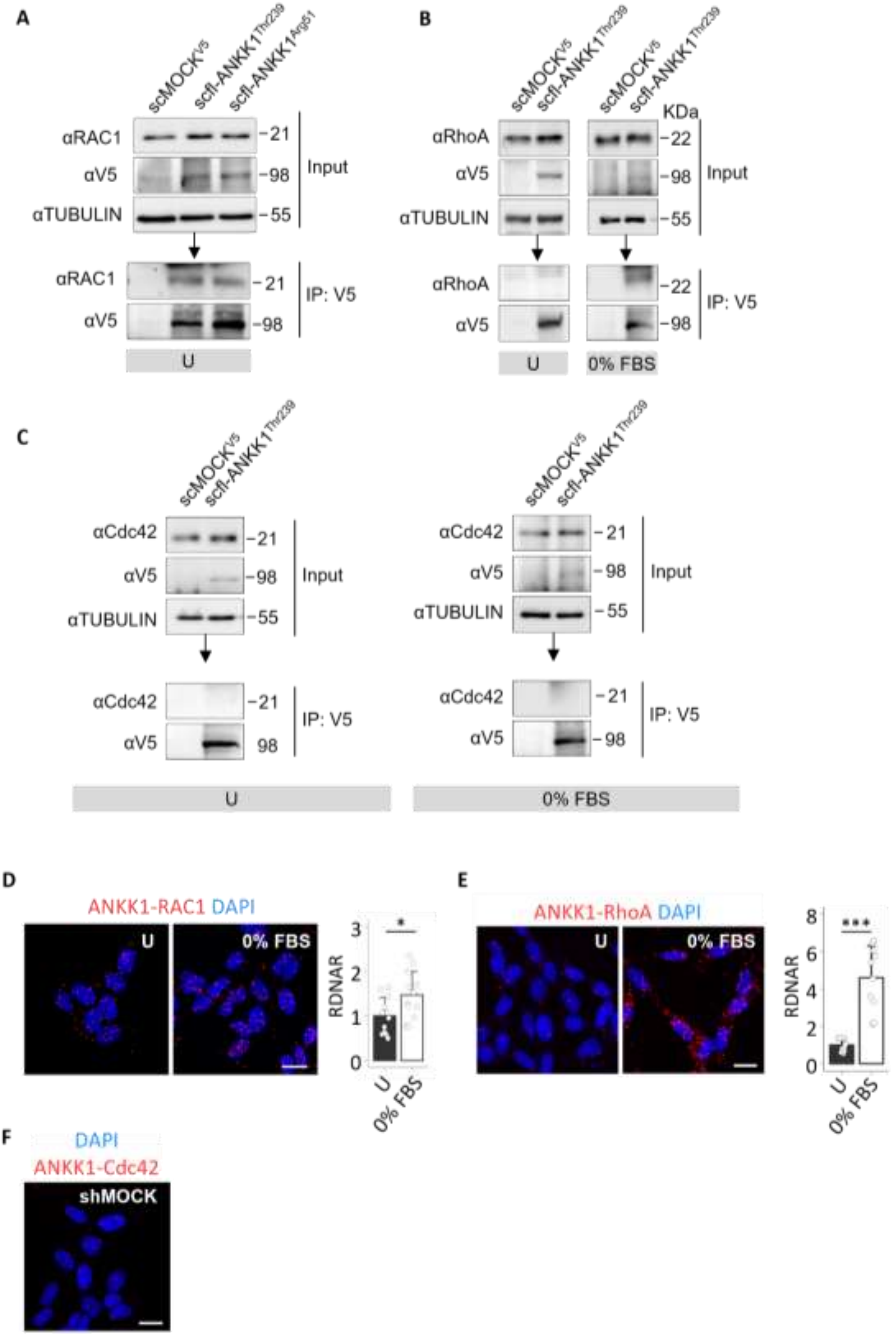

Figure S9. ANKK1 interact with the RhoGTPases RAC1 and RhoA. **A.** Co-IP of endogenous RAC1 and ANKK1 in undifferentiated scMOCK^{V5}, scfl-ANKK1^{Thr239} and scfl-ANKK1^{Arg51} stable cell lines. **B.** Co-IP of RhoA and ANKK1 in undifferentiated (U) or 48 hours serum starved (0%FBS) scMOCK^{V5} and scfl-ANKK1 cell lines. **H.** Representative PLA confocal images showing the interaction of ANKK1–RAC1 (top) or ANKK1–RhoA (below) in proliferative (U) or 48 hours serum-starved (0% FBS) SH-SY5Y cells. **C**. Co-immunoprecipitation of endogenous Cdc42 and ANKK1 in proliferative (U) and 48 hours 0% FBS differentiated SH-SY5Y modified stable cell lines: scMOCK or scfl-ANKK1. Maximum intensity projections are shown. Scale bar: 10 µm. Analysis of RDNAR is shown

in the bar plots below (n=12 images, 3 independent experiments). Plots represent mean±sd and individual values are displayed as dots. (**A, B, H**) One sample *t*-test on the fold-change values was used for MOCK/U-group comparisons. (**C-D**) One sample *t*-test on the fold-change values was used for MOCK-group comparisons. Student's *t*-test on the fold-change values was used for inter-group comparisons. Bonferroni was used as a multiple-testing correction method. (**E**) One sample *t*-test on the fold-change values was used for MOCK(U)-group comparisons. Student's *t*-test (2 groups) or ANOVA (3 groups) on the fold-change values was used for inter-group comparisons. Bonferroni was used as a multiple-testing correction method. **p* < 0.05; ****p* < 0.001. **B.** Representative PLA confocal image showing the interaction of ANKK1 and Cdc42 in shMOCK cells. Maximum intensity projection is shown. Scale bar: 15 µm. Abbreviations: FBS: Fetal bovine serum; U: Undifferentiated.

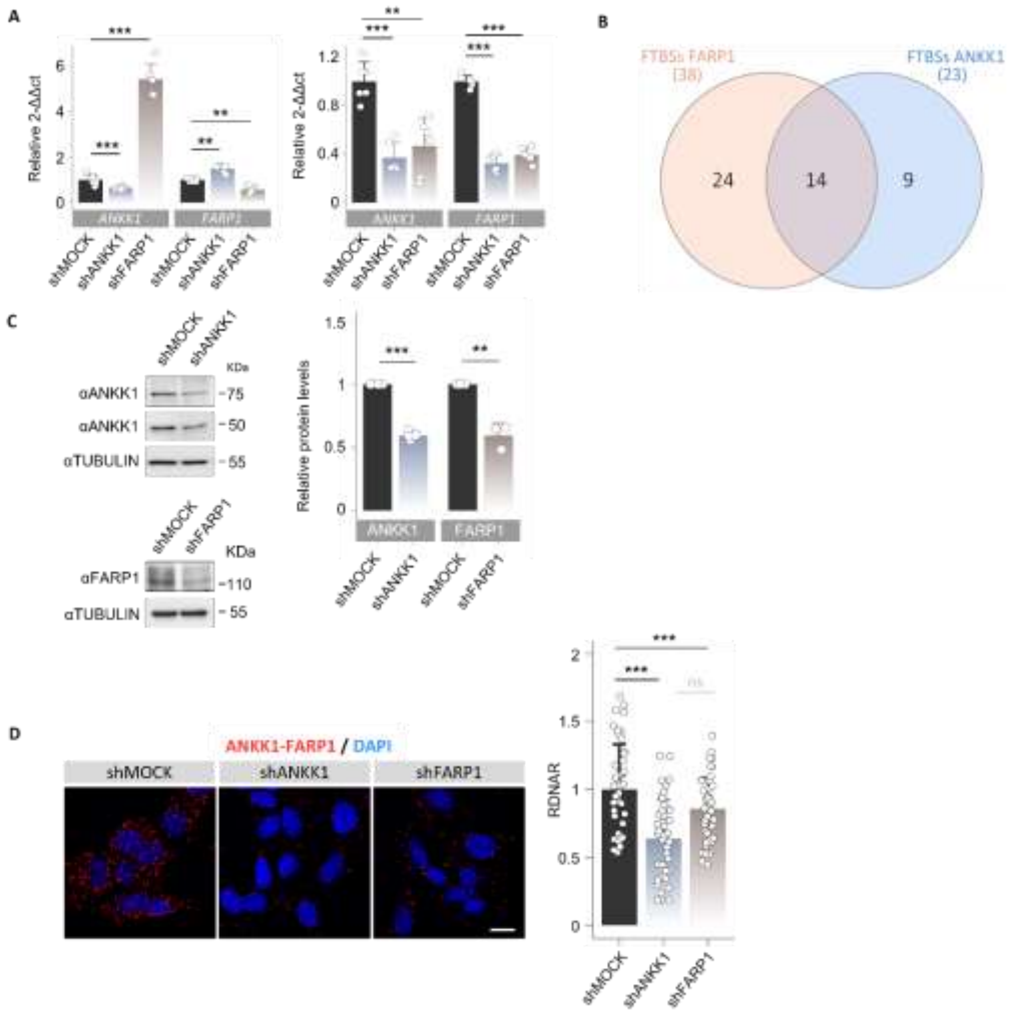

Figure S10. Characterization of *ANKK1* or*FARP1* knockdown SH-SY5Y cell lines. **A**. Quantitative RT-PCR analysis of *ANKK1* and *FARP1* mRNA expression in proliferative (U, left) and after 48 hours 0% FBS differentiated (0%FBS, right) shMOCK, shANKK1 and shFARP1 cell lines (n=4/3 independent experiments, 3 replicates each). **B.** Common TFBS of the promoter regions of *FARP1* and *ANKK1* genes involved in the cell cycle and neural differentiation (see also **Table S1**). **C.** Western blot of ANKK1 and FARP1 in shMOCK, shANKK1, and shFARP1 cell lines. Protein quantification is shown in the adjacent barplot (n=4/3 independent experiments). (**D**) Representative confocal images of the PLA showing the interaction of ANKK1 and FARP1 in proliferative shMOCK, shANKK1, and shFARP1 cell lines. Maximum intensity projections are shown. Scale bar = 15 µm. Analysis of RDNAR is shown in the adjacent bar plot (n=45 images, 3 independent experiments). Barplots represent mean ± SD and individual values are displayed as dots. (**A-C**) One sample *t*-test on the fold-change values was used for MOCK-group comparisons. Bonferroni was used as a multiple-testing correction method. (**D**) One sample *t*-test on the fold-change values was used for MOCK-group comparisons. Bonferroni was used as a multiple-testing correction method. Student's *t* test on the fold-change values was used for between-groups comparison. ***p* < 0.01; ****p* < 0.001. Abbreviations: RDNAR: Relative dots/nuclei area ratio; TFBS: Transcription factor binding sites.

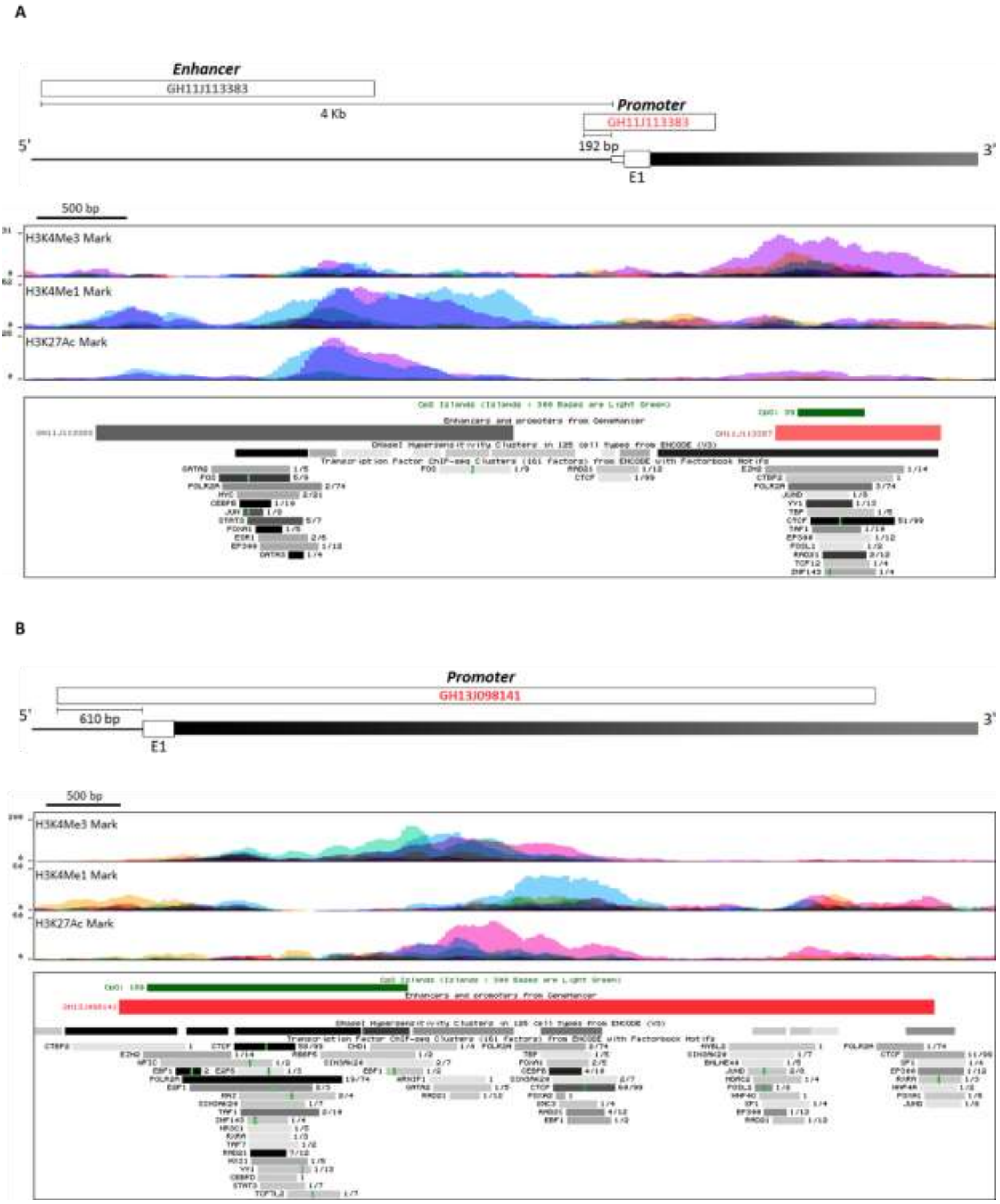

Figure S11. Analysis of *ANKK1* and *FARP1* genes promoters using ENCODE data (GRCh37/hg19). **A. Top:** *ANKK1* upstream region showing both an enhancer located 4 Kb upstream and a promoter 192 bp upstream. **Below:** Chromatin modifications marks of *ANKK1* enhancer GH11J113383 and promoter GH11J113387 in Lymphoblastoid Cell line (GM12878, red), H1 Human Embryonic Stem Cells (H1-hESC, yellow), Human Skeletal Muscle Myoblasts (HSMM, green), Human Umbilical Vein Endothelial Cells (HUVEC, blue), Myelogenous leukemia cells (K562, violet), Normal human epidermal keratinocytes (NHEK, purple) and Normal Human Lung Fibroblasts (NHLF, pink). DNaseI hypersensitivity and CpG Islands indicate regulatory regions and TFBSs. TFBS canonical binding motifs are colored green. **B. Top**: *FARP1* exon 1 and intron 1 regulatory region showing a promoter 610 bp upstream. **Below:** Chromatin modifications marks of *FARP1* promoter GH13IJ098141 in GM12878 (red), H1-hESC (yellow), HSMM (green), HUVEC (blue), K562 (violet), NHEK (purple) and NHLF (pink) cell lines. DNaseI hypersensitivity and CpG Islands indicate regulatory regions and TFBSs. TFBS canonical binding motifs are marked in green. Abbreviations: TFBS: Transcription factor binding sites.

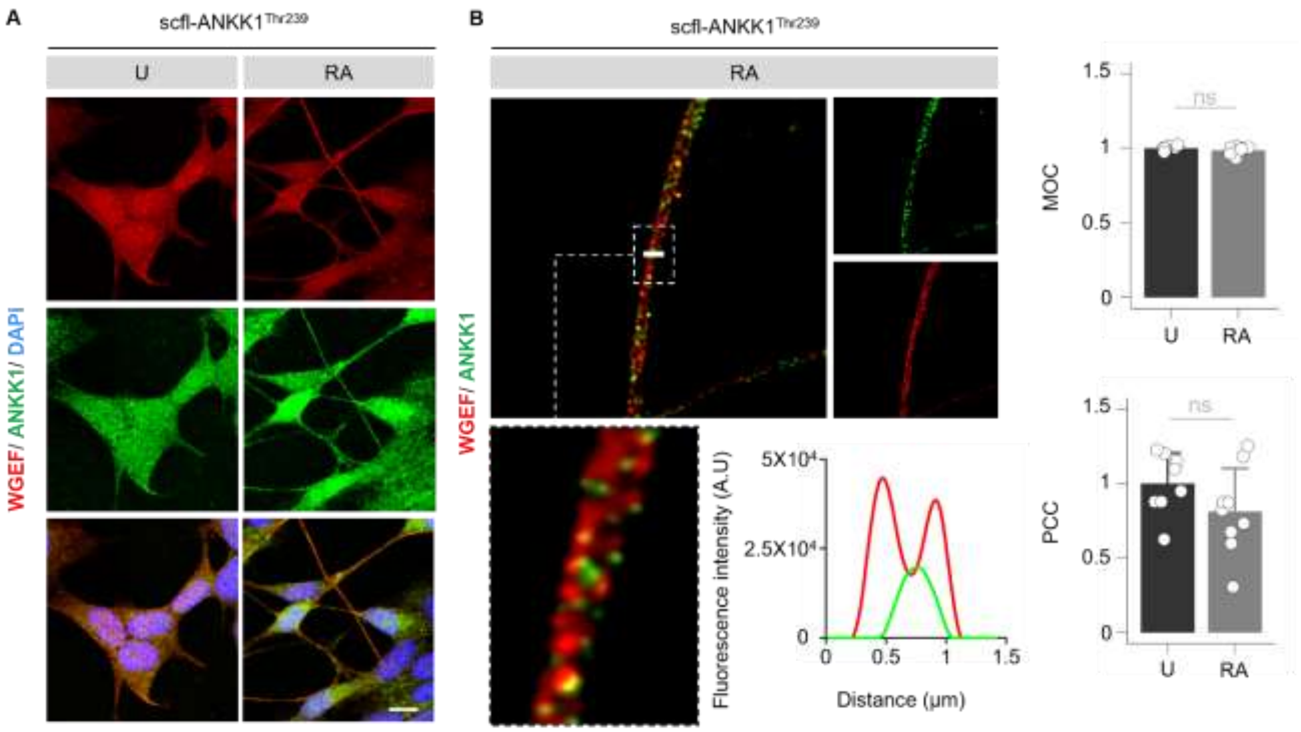

Figure S12. WGEF-ANKK1 interaction in the proliferative scfl-ANKK1 cell line is lost after differentiation induction. **A.** Immunostaining of ANKK1 and WGEF in undifferentiated (U) or RA-treated (RA) scfl-ANKK1^{Thr239} cells (α-V5 and α-WGEF). Maximum intensity projections are shown. Scale bar: 10 µm. **B.** Left: super-resolution stimulated emission depletion (STED) images showing ANKK1 and WGEF localization at neurites in RA-differentiated scfl-ANKK1^{Thr239} cell line. Scale bar: 5 µm. The line profile plots indicate the intensity distribution of the green and red channels along the white straight line crossing the ROI. Right: Quantitative study of ANKK1 and WGEF colocalization by Manders' Overlap Coefficient (MOC) and Pearson correlation coefficient (PCC) in RA-differentiated scfl-ANKK1^{Thr239} cells (n=8/9 images, 3 independent experiments). Plots represent mean±SD and individual values are displayed as dots. One sample *t*-test on the fold-change values was used for MOCK-group comparisons. Student's *t* test or ANOVA on the fold-change values was used for inter-group comparisons. ns: not significant. Abbreviations: A.U: arbitrary units; RA: retinoic acid differentiated cell; ROI: region of interest; U: undifferentiated.

A

ANKK1 HAPLOTYPE	Polimorfisms				
	Kinase Domain	Interdomain	Ankyrin 3	Ankyrin 4	Ankyrin 11
	rs7118900<br>p.Ala239Thr	rs11604671<br>p.Gly318Arg	rs4938016<br>p.Gly442Arg	rs2734849<br>p.His490Arg	rs1800497<br>p.Glu713Lys
**H2B**	G<br>Alanine (=)	G<br>Glycine (=)	G<br>Glycine (=)	T<br>Histidine (+)	C<br>Glutamic acid (-)
**H2**	G<br>Alanine (=)	A<br>Arginine (+)	C<br>Arginine (+)	C<br>Arginine (+)	C<br>Glutamic acid (-)
**H1**	A<br>Threonine (=)	G<br>Glycine (=)	C<br>Arginine (+)	T<br>Hystidine (+)	T<br>Lysine (+)

B

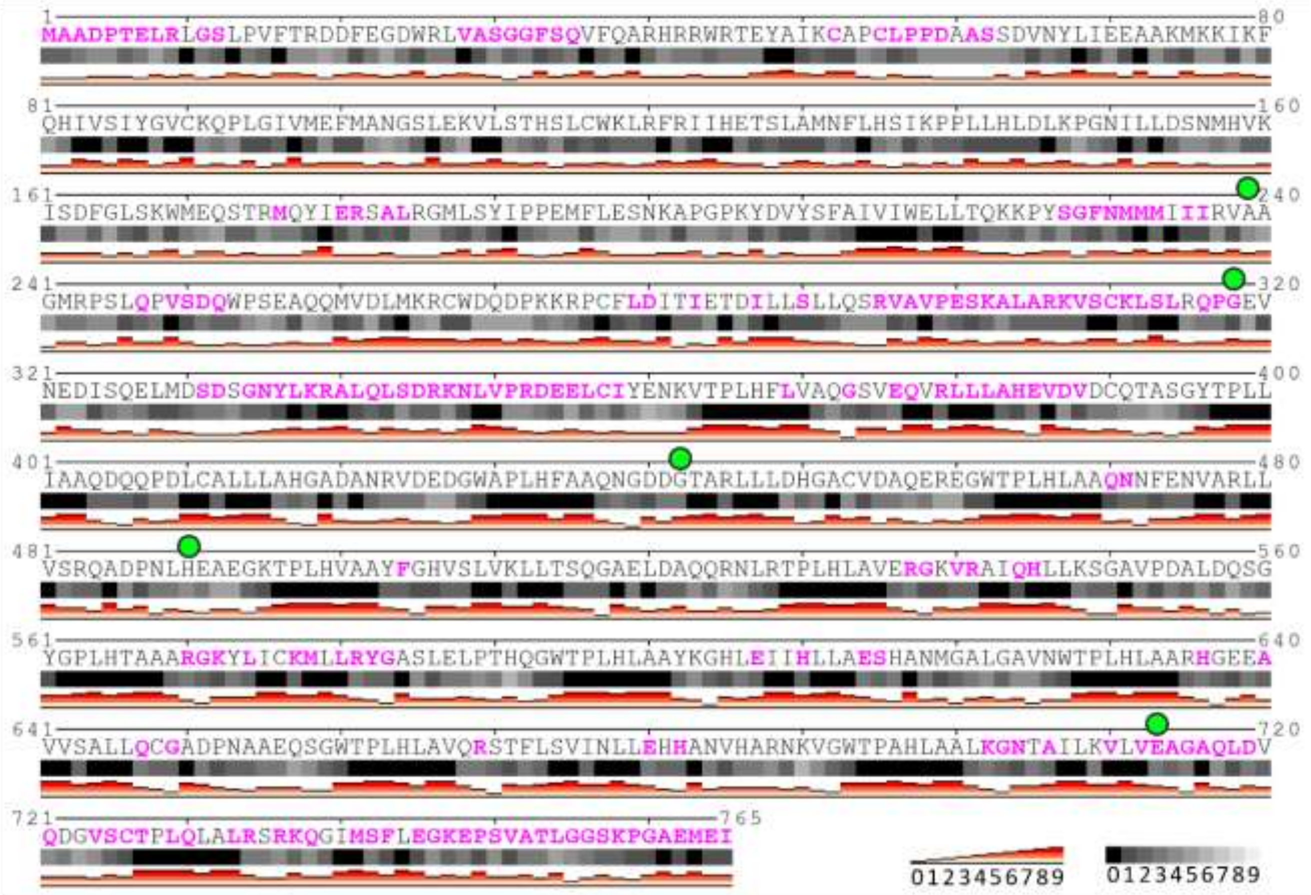

Figure S13. *In silico* analysis of *ANKK1* polymorphic haplotypes and predicted binding sites. **A**. *ANKK1* missense polymorphisms corresponding to haplotypes H2B, H2 and H1. The electric charge for each amino acid is depicted as: (+): positive, (-): negative, (=) neutral. **B**. ANKK1 protein corresponding to haplotype H2B was analyzed using SPPIDER software. Amino acids coloured in pink are predicted binding sites. Predicted binding sites marked in green contain the amino acids positions 318 and 713, which may differ between ANKK1 haplotypes. The prediction confidence level is indicated by a color gradient scale from yellow (lower confidence values) to red (higher confidence values). Relative solvent accessibility is shown in grayscale: from 0 (black), corresponding to a completely buried aminoacid to 9 (white), representing a fully exposed residue.

Supplementary Tables

Table S1. Constructs used and generated in the study

CONSTRUCT	*CONTENT*	*EXPERIMENT/ MAIN FIGURE*
ANKK1^{Thr239}	Thr239 allele of the ANKK1 short isoform in fusion with GFP at the C-terminal end	Neuritogenesis assay (Fig. 2A)<br>Dendritogenesis assay (Fig. 2B)<br>Wound healing assay (Fig. 3B)<br>Relative AP-1 activity (Fig. 4B, C, D, E)
fl-ANKK1^{Thr239}	Thr239-Lys713 allele of the ANKK1 long isoform in fusion with V5 epitope at the N-terminal end	co-IP of overexpressed fl-ANKK1Thr239 and FARP1 (Fig. 1A)<br>Neuritogenesis assay (Fig. 2A)<br>Dendritogenesis assay (Fig. 2B)<br>Wound healing assay (Fig. 3B)<br>Relative AP-1 activity (Fig. 4C, D, E)<br>CoIP of fl-ANKK1^{Thr239}–WGEF (Fig. 6A)
ANKK1^{Ala239}	Ala239 allele of the ANKK1 short isoform in fusion with GFP at the C-terminal end	Relative AP-1 activity (Fig. 4C, D)
fl-ANKK1^{Ala239}	Ala239-Glu713 allele of the ANKK1 long isoform in fusion with V5 epitope at the N-terminal end	Relative AP-1 activity (Fig. 4C, D)
ANKK1^{Arg51}	Thr239 allele of the ANKK1 short isoform in fusion with GFP at the C-terminal end and mutated at lysine 51	Wound healing assay (Fig. 3B)<br>Relative AP-1 activity (Fig. 4E)
fl-ANKK1^{Arg51}	Thr239-Lys713 allele of the ANKK1 long isoform in fusion with V5 epitope at the N-terminal end and mutated at lysine 51	Wound healing assay (Fig. 3B)<br>Relative AP-1 activity (Fig. 4E)
FARP1	FARP1 construct in fusion with GFP at the C-terminal end	Co-IP of overexpressed ANKK1Thr239 and FARP1 (Fig. 1A)<br>Wound healing assay (Fig. 3B)<br>Relative AP-1 activity (Fig. 4A)

Table S2. Modified cell lines generated in the study

CELL LINE	CONTENT	EXPERIMENT/MAIN FIGURE
scANKK1^{Thr239}	SH-SY5Y cell line stably expressing Thr239 allele of the ANKK1 short isoform	Fluorescence intensity signal (Fig. 1F)<br>Neuritogenesis assay (Fig. 2A)<br>Dendritogenesis assay (Fig. 2B)<br>Dendritogenesis assay (Fig. 2B)
scfl-ANKK1^{Thr239}	SH-SY5Y cell line stably expressing Thr239-Lys713 allele of the full lenght ANKK1 isoform	Fluorescence intensity signal (Fig. 1F)<br>Neuritogenesis assay (Fig. 2A)<br>Dendritogenesis assay (Fig. 2B)<br>Dendritogenesis assay (Fig. 2B)
scfl-ANKK1^{Arg51}	SH-SY5Y cell line stably expressing Thr239-Lys713 allele of the full lenght ANKK1 isoform mutated at lysine 51	Co-IP of endogenous RAC1 and scfl-ANKK1^{Arg51} (Fig S9A)
scFARP1	SH-SY5Y cell line stably expressing the FARP1 construct	Fluorescence intensity signal (Fig. 1F) Neuritogenesis assay (Fig. 2A)<br>Dendritogenesis assay (Fig. 2B)
shANKK1	shRNA-mediated ANKK1 knockdown SH-SY5Y cell line	RAC1 and RhoA western blot (Fig. 4F)<br>PLAs of RAC-FARP1 and RhoA-FARP1(Fig. 4G, H)<br>Soma/neurites analysis and F-actin intensity (Fig. 5)<br>PLA RhoA-WGEF (Fig. 6C)<br>WFEG fluorescence intensity (Fig. 6D)
sh-FARP1	shRNA-mediated FARP1 knockdown SH-SY5Y cell line	RAC1 and RhoA western blot (Fig. 4F)<br>PLA of RAC-ANKK1 and RhoA-ANKK1 (Fig. 4G, H)

Table S3. Common TFBS of *ANKK1* and *FARP1* at regulatory genomic elements obtained from ENCODE (GRCh37/hg19).

GENE	PROTEIN	FUNCTION
***ANKK1* and *FARP1* Promoters, and *ANKK1* Enhancer**		
POLR2A	RNA Polymerase II Subunit A	Encodes for the largest subunit of RNA polymerase II, responsible for synthesizing messenger RNA in eukaryotes. Promotes cyclins and cyclin-dependent kinases (CDKs) expression, thereby causing the overall progression of all stages of the cell cycle and **promoting cell proliferation** [8].
P300	E1A Binding Protein P300	Regulates the **proliferation and differentiation of neural stem cells** during adult neurogenesis and regenerative neurogenesis [9]. Under physiological conditions, C646 **(inhibitor of EP300) promoted the proliferation** of neuroendocrine cells and **generation of newborn neurons** [9].
***ANKK1* and *FARP1* Promoters**		
FOXA1	Forkhead Box A1	Has a critical role during embryonic development, and it is activated during retinoic acid (RA)-induced **neural differentiation** [10].
GATA2	GATA Binding Protein 2	Is required for **migration and differentiation of neurons** in the superior colliculus [11]. Cooperatively with GATA3 regulates the differentiation of serotonergic and glutamatergic neuron subtypes of the dorsal raphe [12]. **Regulates negatively the proliferation of neuronal progenitors** [13].
STAT3	Signal Transducer and Activator of Transcription 3	**Promotes neuronal differentiation of embryonic stem** cells and P19 cells in mice while the inhibition of the Stat3 signaling pathway blocks neuronal differentiation in these cells [14] [15].
***ANKK1* Enhancer and *FARP1* Promoter**		
CTCF	CCCTC-Binding Factor	Organizes the linear genome in topologically associating domains (TADs) and loops during the interphase of eukaryotes [16] [17]. Stress-responsive CTCF complexes co-localize with nuclear speckles but, unexpectedly, only at a particular stage of **neuronal differentiation** [18].
CTBP2	C-Terminal Binding Protein 2	CTBP2 inhibition promotes **neuronal differentiation** of neural stem cells [19].
EZH2	Enhancer of zeste homolog 2	Is highly expressed in **proliferating neural stem cells (NSCs)** and its expression decreases during **neuronal differentiation of NSCs** [20].
JUND	AP-1 transcription factor subunit	Is a **negative regulator of cell growth and cell proliferation** [21] [22]. Suppression of JunD protein does not alter the morphological differentiation pattern of p-12 cells suggesting that is not involved in neuronal differentiation [22].
RAD21	RAD21 Cohesin Complex Component	Encodes for double-strand-break repair protein, component of the cohesin complex. Involved in sister chromatid cohesion during mitosis, post-replicative DNA repair [23].

TAF1	TATA-Box Binding Protein Associated Factor 1	Induces cell G1 progression through p53 (a tumor suppressor protein which induces cell cycle arrest and apoptosis in response to DNA damage) [24].
TBP	TATA-Box Binding Protein	Is required for the initiation of transcription by RNA polymerases II. In HepG2 cells induces apoptosis and cell cycle arrest [25].
YY1	Yin Yang-1	Binds to CpG islands at the Sox2 locus and **represses neuron progenitors proliferation** [26]. YY1 knockdown results in increased neurons progenitors and decreased neurons [26].
ZNF143	zinc finger protein 143	Is associated with the CCCTC-binding factor (CTCF) in establishing the conserved chromatin loops by cooperating with cohesin and other partners [27]. It may be involved in the mitosis-to-G1 phase transition [27].

In bold are highlighted the transcription factors associated with neural differentiation.

References

[1] E. Garrido, T. Palomo, G. Ponce, I. Garcia-Consuegra, M.A. Jimenez-Arriero, J. Hoenicka, The ANKK1 protein associated with addictions has nuclear and cytoplasmic localization and shows a differential response of Ala239Thr to apomorphine, Neurotox Res 20(1) (2011) 32-9.

[2] J. Kovalevich, M. Santerre, D. Langford, Considerations for the Use of SH-SY5Y Neuroblastoma Cells in Neurobiology, Methods Mol Biol 2311 (2021) 9-23.

[3] S. Alonso-Orgaz, T. Martin-Rojas, E. Calvo, J.A. Lopez, F. Vivanco, M.G. Barderas, Differential protein expression analysis of degenerative aortic stenosis by iTRAQ labeling, Methods Mol Biol 1005 (2013) 109-17.

[4] J. Hoenicka, A. Quinones-Lombrana, L. Espana-Serrano, X. Alvira-Botero, L. Kremer, R. Perez-Gonzalez, R. Rodriguez-Jimenez, M.A. Jimenez-Arriero, G. Ponce, T. Palomo, The ANKK1 gene associated with addictions is expressed in astroglial cells and upregulated by apomorphine, Biological psychiatry 67(1) (2010) 3-11.

[5] L. Espana-Serrano, N. Guerra Martin-Palanco, A. Montero-Pedrazuela, E. Perez-Santamarina, R. Vidal, I. Garcia-Consuegra, E.M. Valdizan, A. Pazos, T. Palomo, M.A. Jimenez-Arriero, A. Guadano-Ferraz, J. Hoenicka, The Addiction-Related Protein ANKK1 is Differentially Expressed During the Cell Cycle in Neural Precursors, Cereb Cortex 27(5) (2017) 2809-2819.

[6] A. Larionov, A. Krause, W. Miller, A standard curve based method for relative real time PCR data processing, BMC Bioinformatics 6 (2005) 62.

[7] L. Coll, A. Civera-Tregón, J. Hoenicka, F. Palau, B. R., Comparison of different cell segmentation techniques in neuronal cultures, XXXV Annual Congress of the Spanish Society of Biomedical Engineering. Bilbao, Spain, (2017).

[8] Q. Jiang, J. Zhang, F. Li, X. Ma, F. Wu, J. Miao, Q. Li, X. Wang, R. Sun, Y. Yang, L. Zhao, C. Huang, POLR2A Promotes the Proliferation of Gastric Cancer Cells by Advancing the Overall Cell Cycle Progression, Front Genet 12 (2021) 688575.

[9] Y. Shimizu, T. Kawasaki, Histone acetyltransferase EP300 regulates the proliferation and differentiation of neural stem cells during adult neurogenesis and regenerative neurogenesis in the zebrafish optic tectum, Neurosci Lett 756 (2021) 135978.

[10] Y. Tan, Z. Xie, M. Ding, Z. Wang, Q. Yu, L. Meng, H. Zhu, X. Huang, L. Yu, X. Meng, Y. Chen, Increased levels of FoxA1 transcription factor in pluripotent P19 embryonal carcinoma cells stimulate neural differentiation, Stem cells and development 19(9) (2010) 1365-74.

[11] R.T. Willett, L.A. Greene, Gata2 is required for migration and differentiation of retinorecipient neurons in the superior colliculus, The Journal of neuroscience : the official journal of the Society for Neuroscience 31(12) (2011) 4444-55.

[12] M. Haugas, L. Tikker, K. Achim, M. Salminen, J. Partanen, Gata2 and Gata3 regulate the differentiation of serotonergic and glutamatergic neuron subtypes of the dorsal raphe, Development 143(23) (2016) 4495-4508.

[13] A. El Wakil, C. Francius, A. Wolff, J. Pleau-Varet, J. Nardelli, The GATA2 transcription factor negatively regulates the proliferation of neuronal progenitors, Development 133(11) (2006) 2155-65.

[14] M. Snyder, X.Y. Huang, J.J. Zhang, Stat3 is essential for neuronal differentiation through direct transcriptional regulation of the Sox6 gene, FEBS letters 585(1) (2011) 148-52.

[15] J. Pachernik, V. Horvath, L. Kubala, P. Dvorak, A. Kozubik, A. Hampl, Neural differentiation potentiated by the leukaemia inhibitory factor through STAT3 signalling in mouse embryonal carcinoma cells, Folia Biol (Praha) 53(5) (2007) 157-63.

[16] J.R. Dixon, S. Selvaraj, F. Yue, A. Kim, Y. Li, Y. Shen, M. Hu, J.S. Liu, B. Ren, Topological domains in mammalian genomes identified by analysis of chromatin interactions, Nature 485(7398) (2012) 376-80.

[17] E.P. Nora, B.R. Lajoie, E.G. Schulz, L. Giorgetti, I. Okamoto, N. Servant, T. Piolot, N.L. van Berkum, J. Meisig, J. Sedat, J. Gribnau, E. Barillot, N. Bluthgen, J. Dekker, E. Heard, Spatial partitioning of the regulatory landscape of the X-inactivation centre, Nature 485(7398) (2012) 381-5.

[18] B.J. Lehman, F.J. Lopez-Diaz, T.P. Santisakultarm, L. Fang, M.N. Shokhirev, K.E. Diffenderfer, U. Manor, B.M. Emerson, Dynamic regulation of CTCF stability and sub-nuclear localization in response to stress, PLoS genetics 17(1) (2021) e1009277.
[19] L. Zhang, X. Zhang, Y. Zhang, N. Xu, J. Wang, Y. Zhu, C. Xia, Brn4 promotes the differentiation of radial glial cells into neurons by inhibiting CtBP2, Life Sci 254 (2020) 116866.
[20] F. Sher, R. Rossler, N. Brouwer, V. Balasubramaniyan, E. Boddeke, S. Copray, Differentiation of neural stem cells into oligodendrocytes: involvement of the polycomb group protein Ezh2, Stem Cells 26(11) (2008) 2875-83.
[21] C.M. Pfarr, F. Mechta, G. Spyrou, D. Lallemand, S. Carillo, M. Yaniv, Mouse JunD negatively regulates fibroblast growth and antagonizes transformation by ras, Cell 76(4) (1994) 747-60.
[22] K.H. Schlingensiepen, F. Wollnik, M. Kunst, R. Schlingensiepen, T. Herdegen, W. Brysch, The role of Jun transcription factor expression and phosphorylation in neuronal differentiation, neuronal cell death, and plastic adaptations in vivo, Cell Mol Neurobiol 14(5) (1994) 487-505.
[23] H. Cheng, N. Zhang, D. Pati, Cohesin subunit RAD21: From biology to disease, Gene 758 (2020) 144966.
[24] H.H. Li, A.G. Li, H.M. Sheppard, X. Liu, Phosphorylation on Thr-55 by TAF1 mediates degradation of p53: a role for TAF1 in cell G1 progression, Mol Cell 13(6) (2004) 867-78.
[25] G. Ren, J. Hu, Y. Shang, Y. Zhong, Z. Yu, J. An, Tributylphosphate (TBP) and tris (2-butoxyethyl) phosphate (TBEP) induced apoptosis and cell cycle arrest in HepG2 cells, Toxicol Res (Camb) 6(6) (2017) 902-911.
[26] J.L. Knauss, N. Miao, S.N. Kim, Y. Nie, Y. Shi, T. Wu, H.B. Pinto, M.E. Donohoe, T. Sun, Long noncoding RNA Sox2ot and transcription factor YY1 co-regulate the differentiation of cortical neural progenitors by repressing Sox2, Cell Death Dis 9(8) (2018) 799.
[27] B. Ye, G. Yang, Y. Li, C. Zhang, Q. Wang, G. Yu, ZNF143 in Chromatin Looping and Gene Regulation, Front Genet 11 (2020) 338.
